# Model structures for C-(A)-S-H(I)

**DOI:** 10.1107/S2052520614021982

**Published:** 2014-11-08

**Authors:** Ian G. Richardson

**Affiliations:** a School of Civil Engineering, University of Leeds, Woodhouse Lane, Leeds LS2 9JT, England

**Keywords:** cement-based concrete, binding phase, tobermorite

## Abstract

C-(A)-S-H(I) is a calcium silicate hydrate that is studied extensively as a model for the main binding phase in concrete. It is a structurally imperfect form of 14 Å tobermorite that has variable composition and length of (alumino)silicate anions. New structural–chemical formulae are presented for single- and double-chain tobermorite-based phases and equations are provided that can be used to calculate a number of useful quantities from ^29^Si NMR data. It is shown that there are no interlayer calcium ions when the silicate chains are of infinite length and that one is added for each tetrahedral ‘bridging’ site that is vacant. Preparations that have Ca/Si greater than about 1.4 include an intermixed Ca-rich phase. It is not possible to generate a structural model for a dimer that is crystal-chemically consistent with known calcium silicate hydrates if the starting structure is an orthotobermorite, *i.e.* of the type that has been used in all previous studies. Crystal-chemically plausible models are developed that are based instead on clinotobermorite. A number of models that represent different mean chain lengths are developed using crystal-chemical and geometrical reasoning. The models account for experimental observations, including variations in Ca/Si, H_2_O/Si, (alumino)silicate anion structure and layer spacing.

## Abstract 2

C-(A)-S-H(I) is a structurally imperfect form of 14 Å tobermorite that is studied extensively as a model for the main binding phase in concrete. Crystal-chemically plausible structures are developed that account for a wide range of experimental observations, including variations in calcium to silicon ratio, water to silicon ratio, (alumino)silicate anion structure and layer spacing.

## 1. Introduction   

Every year over seven billion cubic metres of Portland cement-based concrete are manufactured worldwide (Gartner, 2004 ▸). The principal binding phase in all of this concrete is a calcium silicate hydrate [C-(A)-S-H^1^] phase. This C-S-H is virtually X-ray amorphous, compositionally and structurally very variable, and generally finely intermixed with other phases, all of which make it difficult to study. Researchers have as a consequence looked for compositional and structural similarity with natural crystalline calcium silicate hydrates – most commonly 14 Å tobermorite (C_5_S_6_H_9_) and jennite (C_9_S_6_H_11_; Taylor, 1986 ▸; Richardson & Groves, 1992*a* ▸) – and have attempted to synthesize single-phase C-S-H in the laboratory that is similar to the phase that forms in concrete. The crystallinity of synthetic C-S-H preparations varies considerably: some have poor powder X-ray diffraction patterns that are similar to those of the C-S-H that is present in most cement pastes, whilst others are quite highly ordered. The latter include C-S-H(I) for Ca/Si ratios less than about 1.4 and C-S-H(II) for higher values. C-S-H(I) has been considered to be a structurally imperfect form of 14 Å tobermorite (Taylor, 1964 ▸, 1997 ▸) and C-S-H(II) to be related in a similar way to jennite (Gard & Taylor, 1976 ▸). Despite much research attention there are currently no structural models available for either phase that can account comprehensively for the experimental observations, which are rather numerous for C-S-H(I). The purpose of this paper is to present a review, collation and new interpretation of the most important data for C-S-H(I) and to provide structural models that account for the observed trends. The models were derived using crystal-chemical and geometrical reasoning, which necessitated a review of general aspects of the crystal chemistry of calcium silicate hydrates and related phases.

## 2. Powder X-ray diffraction data for C-S-H(I)   

C-S-H(I) can be prepared that has a Ca/Si ratio between 2/3 and ≃1.4 by mixing calcium and silicate ions in dilute aqueous suspension at temperatures below ≃ 333 K. Solutions of an alkali silicate and a soluble calcium salt (usually nitrate) are often used or a reactive form of silica is mixed with Ca(OH)_2_ or anhydrous C_3_S or β-C_2_S. An early summary of X-ray powder patterns for C-S-H(I) phases is given by Heller & Taylor (1956 ▸). They noted the following points, which have been modified slightly with information from other early work (Taylor, 1950 ▸; Bernal *et al.*, 1952 ▸; Grudemo, 1955 ▸; Taylor & Howison, 1956 ▸; Kalousek & Prebus, 1958 ▸; Taylor, 1964 ▸, 1969 ▸):

(i) C-S-H(I) can be considered to be a structurally imperfect tobermorite.
(ii) Preparations tend to have a single, broadened basal reflection that has a maximum between 9 and 14 Å; this was interpreted as being due to mixtures of hydrates that have different layer spacings, randomly interstratified in sheets normal to **c**.
(iii) The basal spacing depends on both the Ca/Si and H_2_O/Si ratios.
(iv) The basal spacing decreases from ∼ 13–14 Å at Ca/Si = 0.8 to ∼ 10 Å at Ca/Si = 1.5.
(v) Most other peaks can be assigned *hk*0 indices, but perhaps should be regarded as *hk* band heads.
(vi) There is often a peak at ≃ 5.3 Å that varies markedly in intensity from very, very weak to moderate.
(vii) Diffuse bands at ≃ 2.1 and ≃ 2.4 Å become resolved into separate lines in preparations that are better crystallized.
(viii) Additional reflections can occur, in particular, between 3.2 and 3.6 Å.
(ix) In extreme cases, peaks are only visible at ≃ 3.03–3.07 and ≃ 1.80–1.83 Å, which can be of comparable intensity. This was interpreted as being due to random stratification together with very small crystal size in both the **a** and **c** dimensions.

**Figure 1:**
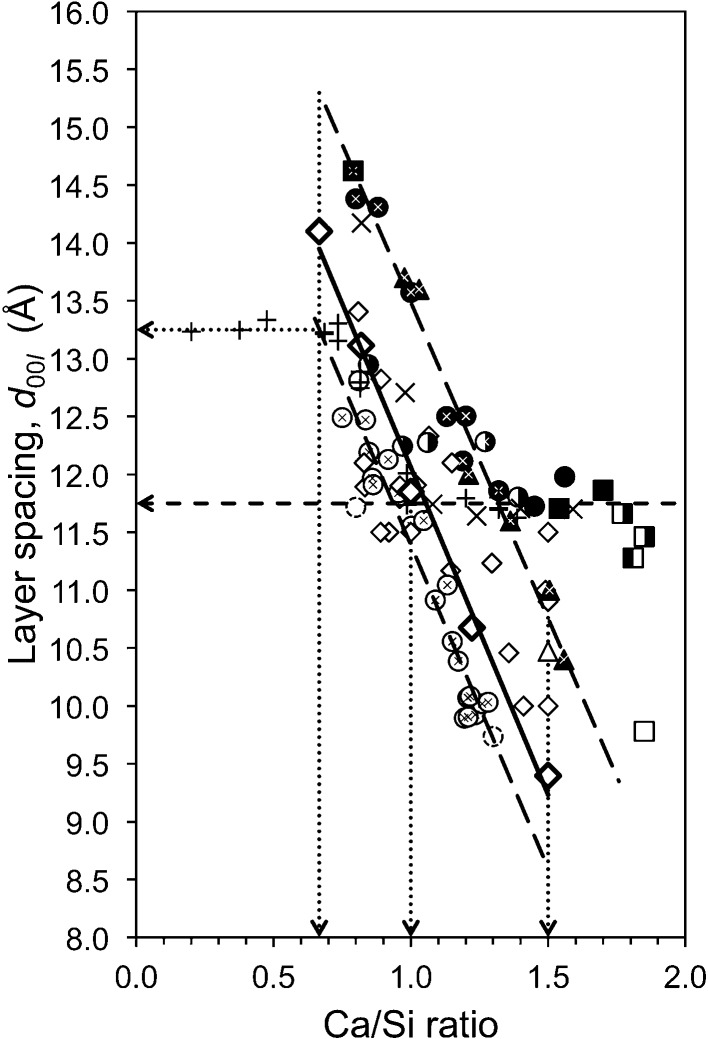
Layer spacing against Ca/Si ratio for C-S-H(I) preparations. Data from: Matsuyama & Young (2000 ▸) (unfilled circles); Matsuyama & Young (1999 ▸) (unfilled dotted-line circles); Cong & Kirkpatrick (1996 ▸) [filled symbols for ‘SCFUM’ and ‘CSHFS’ preparations except ‘CSHSF2’ (unfilled triangle) and CSHFS0 (unfilled square); half-filled (right) symbols for ‘SEWCS’ preparations]; Garbev, Beuchle, Bornefeld, Black & Stemmermann, 2008 ▸ (+), compositions from Black *et al.* (2008 ▸); Renaudin, Russias, Leroux, Frizon & Cau-dit-Coumes (2009 ▸) (crosses); Taylor (1953 ▸) and Taylor & Howison (1956 ▸) (small unfilled diamonds); Grudemo (1955 ▸) (filled triangles). Preparations that contain crystalline Ca(OH)_2_ are indicated with squares except for the filled square at Ca/Si = 0.79, which includes 16% Q^3^ Si. Trend-line data are indicated using white crosses (top line) and black crosses (bottom). The large bold unfilled diamonds represent the model structures developed in this paper; from top to bottom: T∞, T11, T5, T3 and T2.

**Figure 2:**
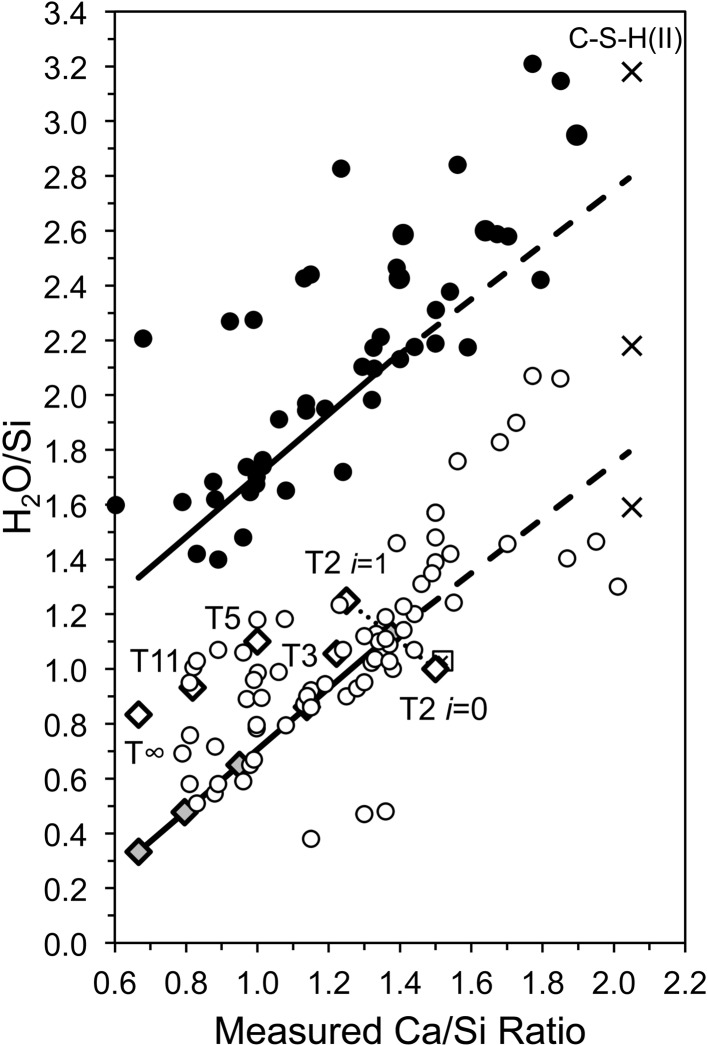
Plot of H_2_O/Si against Ca/Si for C-S-H(I) preparations. The filled circles represent samples that were lightly dried (*e.g.* in flowing N_2_ at room temperature) and the unfilled circles represent samples that were more harshly dried, *i.e.* at around 383 K (or by using a method that is approximately equivalent) or more severely than that [*e.g.* at 509 K and *p*(H_2_O) = 6 mm]. In a few cases data points are in a category different to that reported because it seems likely that they were dried more or less aggressively. The data are from: Brunauer & Greenberg (1962 ▸); Cong & Kirkpatrick (1996 ▸); Copeland *et al.* (1967 ▸); El-Hemaly *et al.* (1978 ▸); Fujii & Kondo (1981 ▸); Gard *et al.* (1959 ▸); Renaudin, Russias, Leroux, Frizon & Cau-dit-Coumes (2009 ▸); Taylor (1950 ▸, 1953 ▸); Taylor & Howison (1956 ▸). Samples with Ca/Si > 1.5 often contained crystalline Ca(OH)_2_. The lower thick line represents equation (23) and the upper black line is the same with one additional water molecule per Si atom. The bold unfilled diamonds represent the model structures that are developed in this paper and the five grey diamonds represent the least hydrated versions of them (from left to right, the T∞, T11, T5, T3 and T2 structures). The long-dashed lines correspond to intermixture of T2(*i* = 0.5) with Ca(OH)_2_. The cross symbols are for C-S-H(II) (Gard & Taylor, 1976 ▸) with different degrees of drying. The crossed-square symbol is for an almost fully reacted bottle-hydrated C_3_S (Brunauer *et al.*, 1958 ▸; Brunauer, 1962 ▸).

These points are still applicable. Grudemo’s (1955 ▸) observation that the basal spacing decreases with increasing Ca/Si ratio has been confirmed by many workers, although the specific results of different investigations differ considerably, which is evident in a recent compilation by Grangeon *et al.* (2013 ▸); see their Fig. 3, which shows much scatter. Grangeon *et al.* (2013 ▸) had apparently missed an earlier compilation by Matsuyama & Young (2000 ▸) (their Fig. 8*a*) who attributed some of the scatter to differences in the degree of sample drying, an explanation that had been noted much earlier by Taylor (1964 ▸). A similar compilation of literature data is given in Fig. 1 ▸, which – following Matsuyama & Young (2000 ▸) – includes two approximately parallel lines; in the present case they are trend lines for the subsets of the data that are marked with black or white crosses. The sources of the data are given in the figure caption; the large bold diamonds (and the associated full line) represent model structures that are developed in §5. On inspection, it is evident that the two lines are separated by ∼ 2 Å over the full range of Ca/Si ratios (*i.e.* 2/3 to 3/2), which – following Matsuyama & Young (2000 ▸) – is interpreted here as being caused by drying, *i.e.* the layer spacing shrinks by ∼ 2 Å because of the removal of water molecules from the interlayer region, the extent of the shrinkage being largely unaffected by the Ca/Si ratio. This interpretation is supported by observations on how the H_2_O/Si ratio is affected by drying. Fig. 2 ▸ is a plot of H_2_O/Si against Ca/Si: the filled circles represent samples that were lightly dried and the unfilled circles represent samples that were dried more harshly. Whilst there is quite a lot of scatter in the data, it is evident from the trend lines that the maximum shrinkage corresponds to the loss of one H_2_O molecule per Si atom; the exact meaning of the full lines is explained in §6.

There are many data points on Fig. 1 ▸ that fall between the two trend lines and also some data that fall to the left of the bottom line or to the right of the top line. Some of the variation could be due to random or systematic errors, either in the determination of the Ca/Si ratio or in the XRD data, such as specimen displacement and zero shift. Certainly some of the studies do not report a method of calibration for the XRD experiments, including Matsuyama & Young (2000 ▸), whose data form the bulk of the points that are clustered around the bottom trend line, whereas the data from Garbev, Beuchle *et al.* (2008 ▸) – who do report careful calibration – fall to the right of this line. It is relevant to this point to note that it is difficult to reconcile the position of the bottom trend line with crystal-chemical arguments (see §5), in particular with the fact that it suggests that the basal spacing is 8.6 Å at Ca/Si = 1.5, which seems unlikely to be possible. In addition to the possibility of experimental errors, the most important explanations are:

(1) The data points with Ca/Si < 2/3 are due to intermixture of C-S-H that has Ca/Si ≥ 2/3 with unreacted silica. The horizontal dotted-line arrow indicates the layer spacing of the C-S-H.
(2) The data points that fall to the right of the upper long-dashed line that have layer spacings between ≃ 11.5 and 12.0 Å are due to intermixture of C-S-H that has Ca/Si ≤ 1.32 with Ca(OH)_2_, evidently either amorphous or crystalline. Intermixture of C-S-H(I) with an amorphous Ca-rich phase has been demonstrated by Richardson *et al.* (2010 ▸) using TEM-EDX. The horizontal dashed-line arrow indicates the layer spacing of the C-S-H.
(3) The data points that fall between the two trend lines are due to either (i) an intermediate level of drying or (ii) to intermixture of a C-S-H that has a Ca/Si that falls on the lower trend line with Ca(OH)_2_, again either amorphous or crystalline.

Explanations (1)[Other l2li1] and (2)[Other l2li2] amount to essentially the same situation as occurs in synthetic layered double hydroxide phases (Richardson, 2013 ▸). Grangeon, Claret, Linard & Chiaberge (2013 ▸) considered that the quite large differences in layer spacing that have been observed for preparations that have the same Ca/Si ratio is due in large part to differences in the number of layers that are stacked in the average crystal. They did however state that the method of synthesis and sample preparation might also be a factor, and Figs. 1 ▸ and 2 ▸ would seem to indicate that the observed variation is adequately explained by differences in the extent of drying or by intermixture of C-S-H(I) with second phases.

A number of workers have used one or more of the published tobermorite structures (Hamid, 1981 ▸; Merlino *et al.*, 1999 ▸, 2000 ▸, 2001 ▸; Bonaccorsi *et al.*, 2005 ▸) as models for whole-pattern or Rietveld-type refinements (Garbev, Beuchle *et al.*, 2008 ▸; Garbev, Bornefeld *et al.*, 2008 ▸; Renaudin, Russias, Leroux, Frizon & Cau-dit-Coumes, 2009 ▸) or for the application of calculations that have been developed specifically for defect structures (Grangeon, Claret, Linard & Chiaberge, 2013 ▸; Grangeon, Claret, Lerouge *et al.*, 2013 ▸). Whilst these studies have provided valuable additional insight into the structure of C-S-H(I) phases, a significant drawback of the methodologies is that none of them attempted to refine the atom positions, essentially because of the lack of information in the diffraction data. The refinements were therefore restricted to the lattice parameters, site occupancy, crystal size, preferred orientation and, in the case of Grangeon, Claret, Lerouge *et al.* (2013 ▸), to examining the implications of the interstratification of layers that have different structure. Unfortunately, crystal-chemical problems will occur in the absence of atom-coordinate adjustment if the lattice parameters are changed by more than only a small amount. As an example, Renaudin, Russias, Leroux, Frizon & Cau-dit-Coumes (2009 ▸) obtained an average Si—O distance of 1.77 Å for a preparation that had Ca/Si = 0.8, which they considered to be an acceptable value for the purpose of their discussion; however, inspection of the structures of known calcium silicate hydrates (see §5) shows that it is in fact an implausibly long distance. This is a particularly serious issue if the C-S-H(I) phase that is of interest has a layer spacing that is significantly larger or smaller than the starting model. An increase or decrease in the *c* parameter results in expansion or compression of the central Ca—O core of the structure and so it is important that this issue is addressed if crystal-chemically sensible distances and coordinations are to be retained in the model structure.

## 3. Structural-chemical formulae for tobermorite-based phases   

As noted in §2, C-S-H(I) is a structurally imperfect form of tobermorite (Taylor, 1964 ▸, 1997 ▸). Tobermorites have layer structures that are classified by the interlayer distance. This distance is represented on powder XRD patterns by a low-angle peak that corresponds to layer thicknesses of approximately 14, 11 and 9 Å (although intermediate spacings also occur), which are associated respectively with the approximate compositions C_5_S_6_H_9_, C_5_S_6_H_5_ and C_5_S_6_H. There are two families of 11 Å tobermorites, which are characterized by orthorhombic or monoclinic subcells. Members of the first family can, as a consequence, be referred to as *ortho*tobermorites and members of the second as *clino*tobermorites (Taylor & Kirkpatrick, 2002 ▸). Approximate parameters for the orthorhombic subcell have been reported as: *a*_s_ = 5.65, *b*_s_ = 3.66, *c*_s_ = 22.6 Å (McConnell, 1954 ▸), space group *Imm*2 (Merlino *et al.*, 1999 ▸; Hamid, 1981 ▸) and for the monoclinic subcell as: *a*_s_ = 5.593, *b*_s_ = 3.645, *c*_s_ = 22.456 Å, β_s_ = 96.97°, space group *I*2/*m* (Hoffmann & Armbruster, 1997 ▸). The structures in both families consist of layers of Ca—O polyhedra that have silicate chains clasped to each side that are kinked to produce a repeat of three tetrahedra, *i.e.* the ‘*Dreierketten*’ conformation in Liebau’s classification (Liebau, 1985 ▸). Additional Ca ions and water molecules occur in an interlayer space. Different types of disorder may occur in 11 Å tobermorite (the subcells correspond to structures in which disorder is complete), which Merlino and co-workers (Merlino *et al.*, 1999 ▸, 2000 ▸, 2001 ▸; Bonaccorsi *et al.*, 2005 ▸; Bonaccorsi & Merlino, 2005 ▸) have explained in terms of the order–disorder (OD) theory of Dornberger-Schiff (1956 ▸). The silicate tetrahedra in the *Dreierketten* that are closest to the Ca—O sheet are called paired tetrahedra (and so abbreviated as ‘PT’), and those that are further away are called bridging tetrahedra (BT). The BT of adjacent layers share an O atom and so the *Dreierketten* are linked, forming double chains that run parallel to **b**. It is the conformation of these *Dreierdoppelketten* that alters the subcell from orthorhombic to monoclinic: the double chains in 11 Å orthotobermorite display 2*mm* symmetry compared with 2/*m* in 11 Å clinotobermorite (Bonaccorsi & Merlino, 2005 ▸). The individual layers in 14 Å tobermorite are too far apart to be linked, and so are single-chain, but the BT of adjacent layers are in any case staggered with respect to one another, the difference corresponding to a shift of *b*/2 in the silicate chains (Bonaccorsi *et al.*, 2005 ▸). Additional interlayer water molecules are present in the expanded interlayer. It is relevant to this paper to note that Henmi & Kusachi (1992 ▸) thought it likely that clino­tobermorite formed at lower temperatures than orthotobermorite (because its ‘*mode of occurrence*’ indicates that it precipitated after orthotobermorite), although it should be noted that Biagioni *et al.* (2012 ▸) observed it after heating a sample of 11 Å orthotobermorite.

Formula (1) is a generalized structural–chemical formula for single-chain tobermorite or C-S-H(I). Aluminium is the main substituent in those phases and so it is included together with charge-balancing ions; Al-substituted C-S-H is referred to as C-A-S-H.
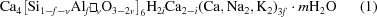
The contents of the square brackets represent the aluminosilicate part of the structure: the □ represents a vacant tetrahedral site; *v* is the fraction of tetrahedral sites that are vacant; *f* is the fraction that are occupied by Al. There are four main-layer Ca atoms for every six tetrahedral sites. The value of *i* reflects the extent to which the net charge is balanced by protons or Ca^2+^ ions. The contents of the round brackets are additional interlayer ions, either monovalent alkali or Ca^2+^ cations, that are needed to charge balance the Al^3+^ substitution for Si^4+^. Formula (1) is very similar to Taylor’s (1993 ▸) formula for purely tobermorite-like structure.

The formula for double-chain tobermorites or, indeed, for cross-linked C-A-S-H phases is
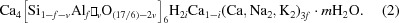
Formulae (1) and (2) can be combined to represent a mixture of single- and double-chain phases
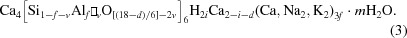
In this formula, *d* represents the fraction of double-chain structure, *i.e.* 0 ≤ *d* ≤ 1, where if *d* = 0 the structure is entirely single chain, and if *d* = 1 it is entirely double chain. These formulae and the equations that are given below are applicable to C-A-S-H phases in general.

**Figure 3:**
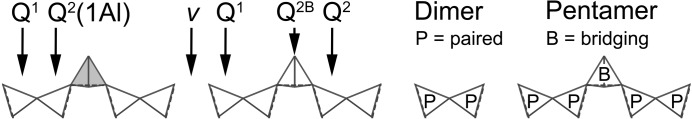
Schematic diagram that illustrates the nature of the linear aluminosilicate chains in C-S-H. The unfilled triangles represent Si—O tetrahedra and the shaded triangle represents an Al—O tetrahedron. Q^2^ = Q^2P^; Q^2^ is sometimes written as Q^2^(0Al) to indicate the lack of Al at one of the two adjacent tetrahedral sites. *v* = vacant tetrahedral site.

Values for the variables in formulae (1)–(3) [Disp-formula fd2] [Disp-formula fd3] can be determined by experiment, although in practice problems can arise where samples contain more than one phase. ^29^Si MAS NMR can be used to provide quantitative information on the fractions of silicon that are present in different tetrahedral environments because an increase in polymerization results in characteristic up-field ^29^Si chemical shifts. These shifts are further influenced by the replacement of Si by Al. There are 15 possible Q^*n*^(*m*Al) structural units where the silicate tetrahedron, Q, is connected *via* oxygen bridges to *m* Al and *n* − *m* other Si atoms, with *n* = 0 to 4 and *m* = 0 to *n*. The Q tetrahedra that are present in single-chain tobermorites or C-A-S-H phases are illustrated in Fig. 3 ▸. Double-chain tobermorites also have Q^3^(0Al) and Q^3^(1Al) sites. Expressions are given in Table 1 ▸ for the fractions of the tetrahedral sites in tobermorite and C-A-S-H phases that are occupied by Si or Al or that are vacant. It is assumed that Al only substitutes for Si at bridging sites (Richardson & Groves, 1993*a* ▸), that Al cannot occur at adjacent bridging sites and that paired sites cannot be vacant. The assumption that Al cannot substitute for Si at paired sites is well supported by a number of experimental studies on single-chain structures (Richardson, Brough *et al.*, 1993 ▸; Richardson, 1999 ▸; Andersen *et al.*, 2003 ▸; Sun *et al.*, 2006 ▸).

The fraction of vacant tetrahedral sites that are present in double- or single-chain tobermorite or C-A-S-H can be calculated from ^29^Si NMR data using equation (4).
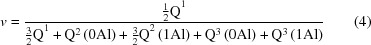
In this equation – and those that follow – Q*^n^*(*m*Al) represents the relative intensity of a peak determined from the deconvolution of a ^29^Si single-pulse MAS NMR spectrum (assuming that the spectrum was collected using quantitative conditions). Q^3^(*m*Al) = 0 when the structure is single chain.

The mean aluminosilicate chain length (MCL) can be calculated using
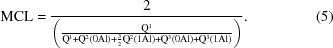
In the case of double chains, MCL corresponds to the average number of linked tetrahedra in sections of the chains along **b** that are separated by vacant sites, and thus includes paired Q^1^ and Q^2^ tetrahedra, and bridging Q^2^ and Q^3^ tetrahedra, the Q^3^ being cross-linked with other bridging tetrahedra. Again, Q^3^(*m*Al) = 0 for single-chain tobermorite and in that case the expression reduces to that given by Richardson *et al.* (1994 ▸).

The MCL and the fraction of vacant tetrahedral sites, *v*, are related simply by
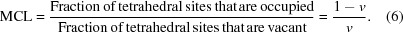
So for example, since one third of all tetrahedral sites are bridging sites, if all of the bridging sites were vacant, 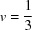 and 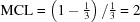; *i.e.* the chains would be entirely dimeric. If one half of the bridging sites were vacant, then 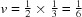 and 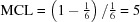; *i.e.* the chains would on average be pentameric. Whilst such short chains are not relevant to the crystalline tobermorite phases, they are relevant to the C-S-H(I) phases and also to tobermorite-based models for the C-S-H that forms in hardened cements. The fraction of vacant tetrahedral sites can therefore be calculated from the MCL using
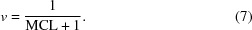
The fraction of tetrahedral sites that are occupied by Al can be calculated from ^29^Si NMR data using
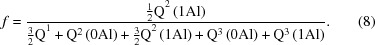
The Al/Si ratio can be calculated using
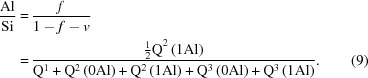
Q^3^(*m*Al) = 0 for single-chain tobermorite and so in that case the expression reduces to that given by Richardson *et al.* (1994 ▸) for C-A-S-H. The Q^3^(1Al) is not included in the numerator because all of the Al is accounted for by the Q^2^(1Al).

The site occupancy factor for the bridging site is
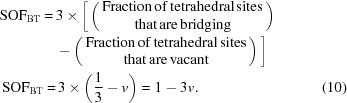



If Al ions are present at the bridging site as well as Si, then site occupancy factors for Si and Al are
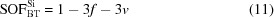


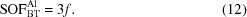
Values for SOF_BT_ can be calculated from NMR data by first calculating *v* and *f* using equations (4) and (8) and then substituting these into equations (10)–(12) [Disp-formula fd11] [Disp-formula fd12]. Such values should be used to inform refinements of XRD data for single-phase tobermorite or C-A-S-H(I).

From formula (1) the Ca/Si ratio of Al-free C-S-H(I) is
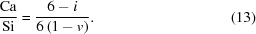
Also, since the maximum Ca/Si ratio is obtained when the net charge is balanced entirely by Ca^2+^ ions, *i.e.* when *i* = 0
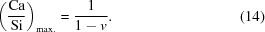
This means that when compositional variation in a single-chain tobermorite-based structure occurs by the omission of some or all of the bridging tetrahedra and by variation in the content of interlayer Ca ions, the maximum Ca/Si ratio that is possible is numerically equal to the number of tetrahedral sites per occupied site. It can therefore be calculated easily from ^29^Si NMR data by substituting for *v*.

## 4. Silicate anion structure of C-S-H(I)   

**Figure 4:**
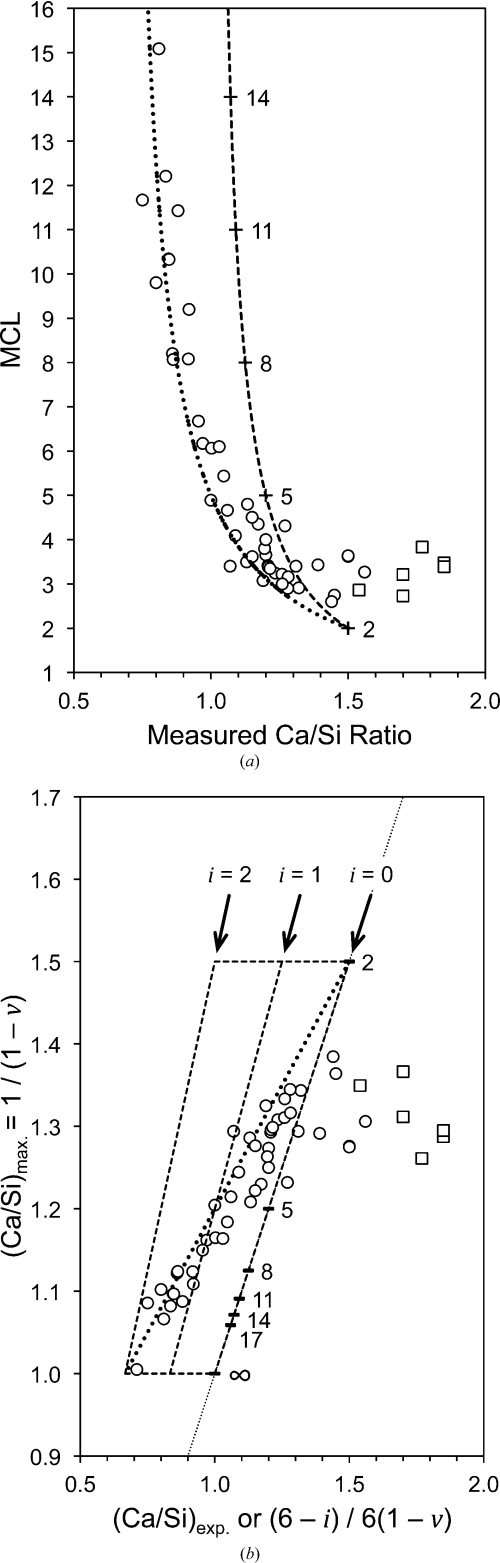
(*a*) MCL and (*b*) 1/(1 − *v*) against the bulk Ca/Si ratio of the C-S-H(I) preparation. The data are from Chen *et al.* (2004 ▸), Cong & Kirkpatrick (1996 ▸), Damidot *et al.* (1995 ▸), Grutzeck *et al.* (1989 ▸), Matsuyama & Young (2000 ▸) and Nonat & Lecoq (1998 ▸). The dashed line in (*a*) and the furthest-right dashed line in (*b*) correspond to the negative charge of the silicate anions balanced entirely by Ca^2+^ ions. The dotted line in both parts of the figure represents equation (15).

C-S-H(I) preparations that are free of Al [*i.e. f* = 0 in formula (1) have been studied extensively using ^29^Si MAS NMR (*e.g.* Cong & Kirkpatrick, 1996 ▸; Grutzeck *et al.*, 1989 ▸; Rassem, Zanni-Theveneau, Vernet, Grimmer *et al.*, 1992 ▸; Rassem, Zanni-Théveneau, Vernet, Heidemann *et al.*, 1992 ▸; Damidot *et al.*, 1995 ▸; Klur *et al.*, 1998 ▸; Nonat & Lecoq, 1998 ▸; Lequeux *et al.*, 1999 ▸). Figs. 4 ▸(*a*) and (*b*) show values of MCL and 1/(1 − *v*) (*i.e.* the maximum theoretical Ca/Si ratio) calculated using data from several sources plotted against the experimental bulk Ca/Si ratio (the sources are given in the figure caption). Data for preparations that have Q^3^ tetrahedra or unreacted silica are not included and those that contain crystalline Ca(OH)_2_ are represented using square symbols. The positions for silicate chains of finite length are indicated; *i.e.* of length 3*n* − 1, where *n* is an integer. It is evident from Fig. 4 ▸(*a*) that the MCL decreases quickly from ≃ 11 at a Ca/Si ratio of 0.8 to ≃ 5 at Ca/Si of 1.0 and then slowly to ≃ 3 at Ca/Si of ∼ 1.2, where it remains upon further increase in the Ca/Si. The maximum Ca/Si ratio that is possible without the presence of Ca—OH linkages in the C-S-H structure, (Ca/Si)_max_ [*i.e.* 1/(1 − *v*)], is represented by a short-dash line in Fig. 4 ▸(*a*). It corresponds to the situation where the negative charge of the silicate anions is balanced entirely by Ca^2+^ ions; Si—OH linkages must be present if a point is to the left of this line and Ca—OH linkages must be present if a point is to the right of it. The imbalance of charge therefore requires the presence of Si–OH at low Ca/Si ratios and Ca—OH at high ratios, with neither needed at ≃ 1.3. In addition to the line for (Ca/Si)_max_ (*i.e. i* = 0), Fig. 4 ▸(*b*) also has lines for *i* = 1 and 2. These two lines correspond respectively to the negative charge being balanced equally by protons and Ca^2+^ ions and totally by protons. The area bounded on Fig. 4 ▸(*b*) by the short-dash lines thus encompasses all of the compositions that are possible for single-chain tobermorite-based structures when the structural variation is the omission of some or all of the bridging tetrahedra and variation in the content of interlayer Ca ions. This is just an alternative way of representing the information in Fig. 3 ▸ of Richardson & Groves (1992*a* ▸) [which has 1/MCL plotted against Ca/Si instead of 1/(1 − *v*), which is (MCL + 1)/MCL]; the line for *i* = 1 is also therefore equivalent to the line for tobermorite-based structure in Fig. 2 ▸ of Taylor (1986 ▸). It is evident that the data for these synthetic C-S-H(I) preparations broadly follow the diagonal dotted line that is plotted on Fig. 4 ▸(*b*) from *i* = 2 at low Ca/Si ratio to *i* = 0 at high ratio. This line represents the equation
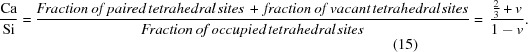
The fraction of tetrahedral sites in tobermorite-based structures that are ‘paired’ is numerically the same as the number of main-layer Ca atoms per three tetrahedral sites [*i.e.* 2/3; as shown in formula (1)]. The dotted line therefore represents the situation where one Ca^2+^ ion is added to the interlayer region for each bridging site that is vacant, and there are consequently no interlayer calcium ions when the chains are of infinite length. This situation is also represented by a dotted line on Fig. 4 ▸(*a*). On inspection, it can be seen that if expressed in terms of six tetrahedral sites, equation (15) is the same as equation (13) with *i* = 2 − 6*v*; *i.e. i* = 2 for infinite chains (*v* = 0) and *i* = 0 for dimeric chains (*v* = 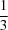).

**Figure 5:**
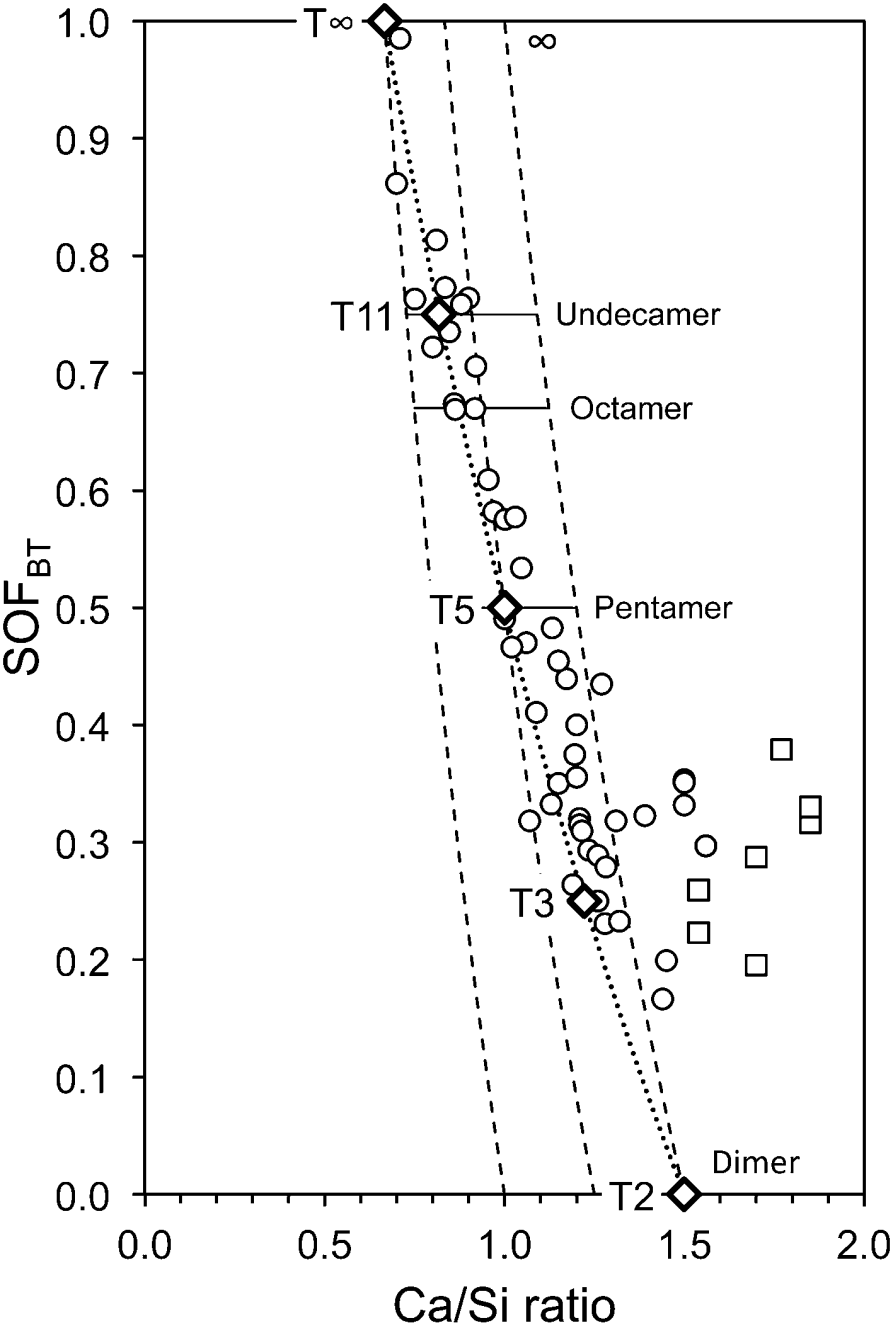
Plot of SOF_BT_ against bulk Ca/Si ratio for C-S-H(I) preparations. The data are from: Chen *et al.* (2004 ▸); Cong & Kirkpatrick (1996 ▸); Damidot *et al.* (1995 ▸); Grutzeck *et al.* (1989 ▸); Matsuyama & Young (2000 ▸); Nonat & Lecoq (1998 ▸); Brunet *et al.* (2004 ▸); García-Lodeiro *et al.* (2012 ▸). Square symbols indicate preparations that contain crystalline calcium hydroxide. The bold unfilled diamonds represent the model structures that are developed in this paper. The three dashed lines represent from left to right, *i* = 2, 1 and 0, respectively. The dotted line represents equation (16), *i.e. i* = 2 × SOF_BT_.

The variation in the silicate anion structure that is represented by equation (15) is perhaps more easily envisaged if the equation is recast in terms of SOF_BT_. Substitution for *v* in equation (15) results in equation (16), which can be used to calculate the Ca/Si ratio from single-pulse ^29^Si NMR data. The data in Fig. 4 ▸ are replotted in Fig. 5 ▸ in terms of SOF_BT_, together with a line that represents equation (16) (the dotted line), which – given the various errors that are possible in the experimental data – is evidently a good explanation for most of the data, which as a consequence provides a useful constraint for the development of model structures.
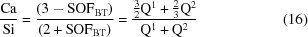
Brunet *et al.* (2004 ▸) studied ^29^Si-enriched C-S-H(I) samples using ^29^Si double quantum homonuclear CP/MAS correlation NMR experiments and observed strong Q^1^–Q^1^ correlation even in a sample that had a bulk Ca/Si ratio of 0.9. This is significant because it means that there must have been some dimeric structure, even at the low Ca/Si ratio of 0.9, which is consistent with the conclusions from Cong & Kirkpatrick’s (1996 ▸) ^1^H–^29^Si cross-polarization experiments. Any model or models for the structure of C-S-H(I) must therefore account for dimeric structure over most of the compositional range, *i.e.* Ca/Si from 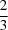 up to about 1.5.

The data that are compiled in this section are consistent with the view that C-S-H(I) with Ca/Si less than about 1.4 has a structure that is derived from 14 Å tobermorite and that preparations that have Ca/Si greater than about 1.4 include a Ca-rich phase intermixed with the C-S-H(I). The resulting structural–chemical formula is compatible with many of the models that have been proposed for the C-S-H that forms in cement pastes (for this range of composition), as discussed by Richardson (2004 ▸, 2008 ▸) and which is demonstrated in Table 2 ▸.

## 5. Tobermorite-based structural models for Ca/Si ratios up to 1.5   

### 5.1. General aspects of the crystal chemistry of calcium silicate hydrates and related phases   

**Figure 6:**
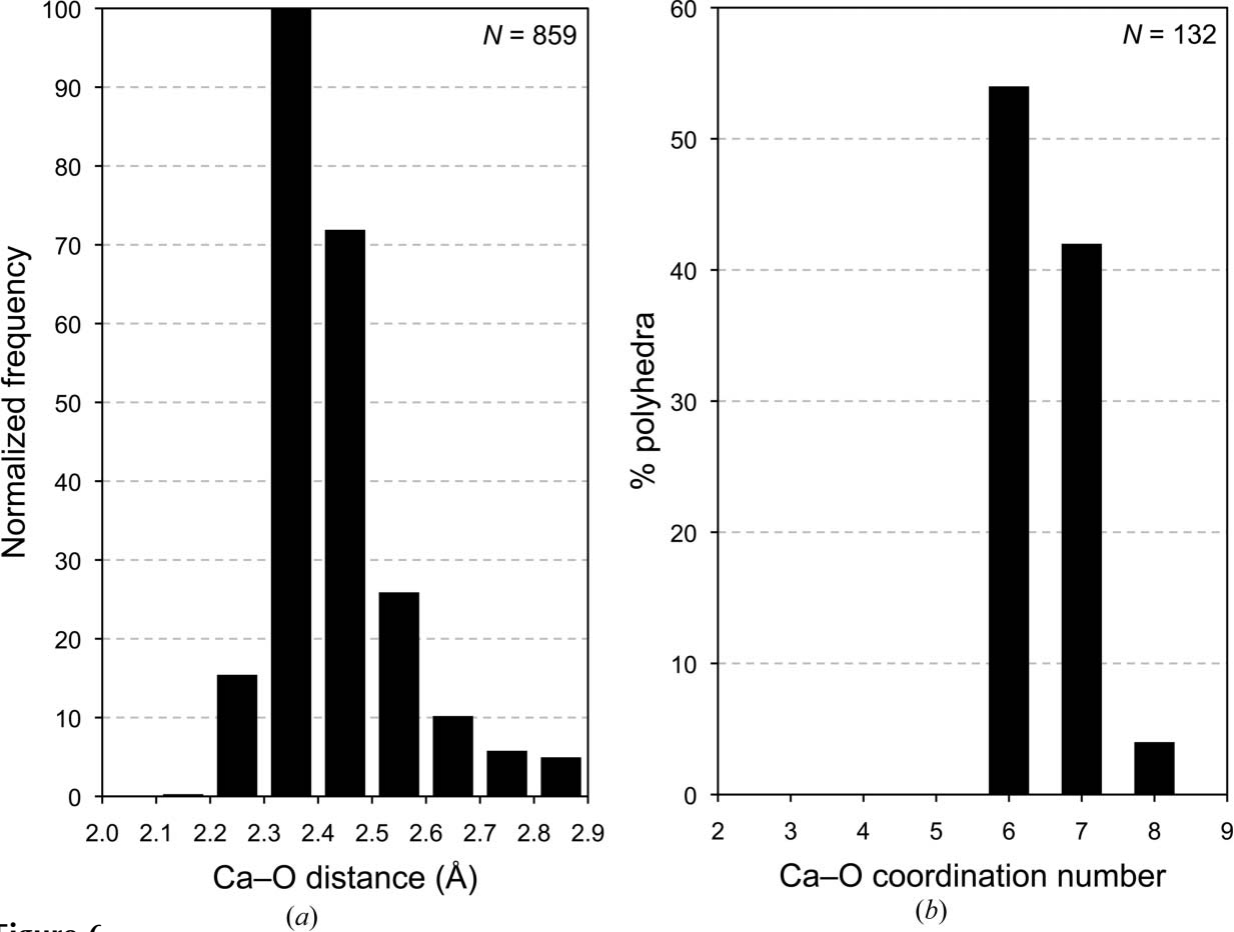
(*a*) Normalized frequency histogram for the Ca—O distances that are present in the crystal structures of 35 crystalline calcium silicate hydrates and related phases. (*b*) Histogram that shows the percentage of the Ca atoms in these phases that are coordinated to different numbers of O atoms. The data include: 11 Å tobermorite (anomalous; MDO_2_ polytype; Merlino *et al.*, 1999 ▸, 2000 ▸); 11 Å tobermorite (normal; MDO_2_ polytype; Merlino *et al.*, 2001 ▸); 14 Å tobermorite (MDO_2_ polytype; Bonaccorsi *et al.*, 2005 ▸); 9 Å clinotobermorite (MDO_2_ polytype; Merlino *et al.*, 1999 ▸, 2000 ▸); afwillite (Malik & Jeffery, 1976 ▸); bultfonteinite (McIver, 1963 ▸; Biagioni *et al.*, 2010 ▸); calcium chondrodite (Kuznetsova *et al.*, 1980 ▸); chegemite (Galuskin *et al.*, 2009 ▸); clinotobermorite (MDO_2_ polytype; Merlino *et al.*, 1999 ▸, 2000 ▸); cuspidine (Saburi *et al.*, 1977 ▸); dellaite (Safronov *et al.*, 1981 ▸); fedorite (Mitchell & Burns, 2001 ▸); foshagite (Gard & Taylor, 1960 ▸); fukalite (Merlino *et al.*, 2009 ▸); gyrolite (Merlino, 1988 ▸); hillebrandite (Dai & Post, 1995 ▸); jaffeite (Yamnova *et al.*, 1993 ▸); jennite (Bonaccorsi *et al.*, 2004 ▸); K-phase (Gard *et al.*, 1981 ▸); kilchoanite (Taylor, 1971 ▸); killalaite (Taylor, 1977 ▸); nekoite (Alberti & Galli, 1980 ▸); okenite (Merlino, 1983 ▸); pectolite (Takéuchi & Kudoh, 1977 ▸); poldervaartite (Dai *et al.*, 1993 ▸); reyerite (Merlino, 1988 ▸); rosenhahnite (Wan *et al.*, 1977 ▸); rustumite (Howie & Ilyukhin, 1977 ▸); scawtite (Zhang *et al.*, 1992 ▸; cell parameter *a* given in the paper as 10.0394 is a mistake and has been corrected to 11.0394 Å); suolunite (Ma *et al.*, 1999 ▸); tilleyite (Grice, 2005 ▸); wollastonite 1 A (Ohashi, 1984 ▸); xonotlite (Hejny & Armbruster, 2001 ▸; polytype derived from a structure in Kudoh & Takeuchi, 1979 ▸); Z-phase (Garbev, 2003 ▸; model structure); α-C_2_SH (Marsh, 1994 ▸).

Any model for the structure of C-S-H(I) should be crystal-chemically plausible. Some general crystal-chemical principles can be established by inspection of the structures of related phases for which crystal structures have been reported. Examination of the structures of 35 crystalline calcium silicate hydrates and related phases (that have a total of 132 unique Ca atoms) shows that out of 859 Ca—O distances there is only one that is < 2.2 Å, as shown in Fig. 6 ▸(*a*). There are only five Ca—O distances that are > 2.9 Å and three of those are in one polyhedron in the interlayer of clinotobermorite (three long weak Ca—O bonds and five shorter, strong bonds), so such long bonds are very rare and 2.9 Å can be taken as a reasonable maximum Ca—O bond length. It is evident that 2.9 and 2.2 Å can be considered to be the maximum and minimum values in structural studies of calcium silicate hydrates. Inspection of the structures of the 35 crystalline calcium silicate hydrates using these values reveals that the ‘natural’ coordination for calcium cations in these phases is either six- or sevenfold, which is illustrated in Fig. 6 ▸(*b*). There are a small number of Ca atoms coordinated to eight oxygen atoms, but none to fewer than six, which is an important observation for atomistic modelling studies that are concerned with C-S-H. For example, as noted by Richardson (2013 ▸), more than half of the Ca atoms in the model of Pellenq *et al.* (2009 ▸) (which has 99 unique Ca atoms) are coordinated to fewer than six O atoms (their model has Ca in five-, four- and even threefold coordination), which is evidently implausible.

**Figure 7:**
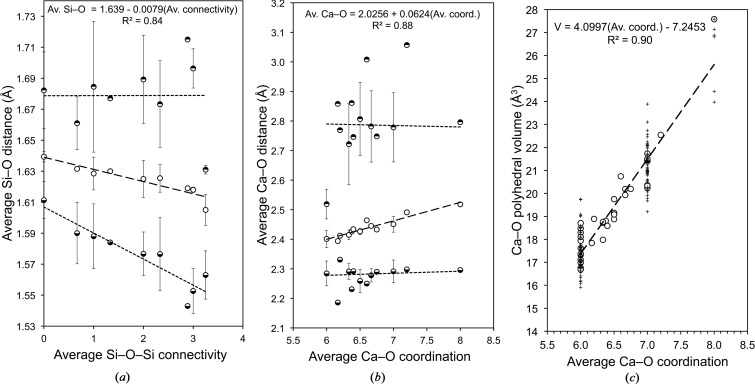
Results from the analysis of the crystal structures of 35 crystalline calcium silicate hydrates and related phases (the references are given in the caption to Fig. 6 ▸): (*a*) the average Si—O distance in the silicate tetrahedra plotted against average Si—O—Si connectivity; (*b*) the average Ca—O distance in Ca—O polyhedra plotted against the coordination number; (*c*) the Ca—O polyhedral volume plotted against the average Ca—O coordination number [values for individual polyhedra (+) and average values for phases (circles)].

Fig. 7 ▸(*a*) shows average Si—O distances in the silicate tetrahedra present in the 35 phases plotted against average Si—O—Si connectivity. There are three sets of points for each value of connectivity: the middle set shows the average of the average distance present in the phases; the upper set represents the average of the maximum distance; and the lower set represents the average of the minimum distance. It is evident that the average Si—O distance in a silicate tetrahedron present in a calcium silicate hydrate decreases linearly with increasing connectivity of the tetrahedron. The linear correlation is quite good (*R* ^2^ = 0.84) and so the regression equation [equation (17)] can therefore be used as a reasonable guide in structural and atomistic modelling studies that are concerned with such phases.
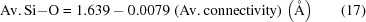
The average value of the minimum distance decreases rather more rapidly than that of the average distance, whilst the average of the maximum distance is essentially constant, although there is much scatter in the data. These data indicate that the silicate tetrahedra in calcium silicate hydrates become increasingly distorted as the connectivity of the tetrahedra increases.

Fig. 7 ▸(*b*) shows that for the Ca—O polyhedra that are present in the 35 phases, there is a good linear correlation between the average Ca—O distance and the average Ca—O coordination; the linear regression equation is given in equation (18) (*r* ^2^ = 0.88), which should provide a useful guide for modelling studies, as indeed should the maximum and minimum values.
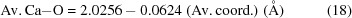
A good linear correlation also exists between the volume of the Ca—O polyhedra and the average Ca—O coordination number [Fig. 7 ▸ *c*, equation (19); *r* ^2^ = 0.90].
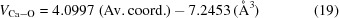



### 5.2. Structural models for C-S-H(I) derived using crystal-chemical and geometrical reasoning   

It is noted in §2 that refinements that involve even quite small changes in lattice parameters can lead to crystal-chemically implausible distances and coordinations if atomic coordinates are not included in the refinement, which has so far proved to be impossible because of the lack of information in the diffraction data. The problem with a change in the *c* parameter resulting in expansion or compression of the central Ca—O core of the structure is resolved in this work by recalculation of the *z*/*c* coordinate of the atoms in the asymmetric unit so that the relative *z* positions of the atoms in the central Ca—O core are unaffected by the change.

The details of the 16 model structures that are developed in this section are deposited^2^ in a CIF file. The name of the datablock for each structure starts with ‘T’ to denote ‘tobermorite-based structure’; a number or ‘∞’ that denotes the mean length of the silicate chains; and an underscore. The rest of the datablock name can be understood after reading §5 and using the following key: 11 or 14 = any infinite-chain part of the model structure has a layer spacing of approximately 11 or 14 Å; s = staggered; a = adjacent; o = *ortho*; c = clino; noCa = there are no interlayer Ca^2+^ ions; LS1 = the first of two alternative layer spacings; LS2 = the second of two alternative layer spacings. Datablock names from the CIF are referred to throughout the text and figure captions.

#### 5.2.1. Dimeric structures that are based on ortho­tobermorite   

**Figure 8:**
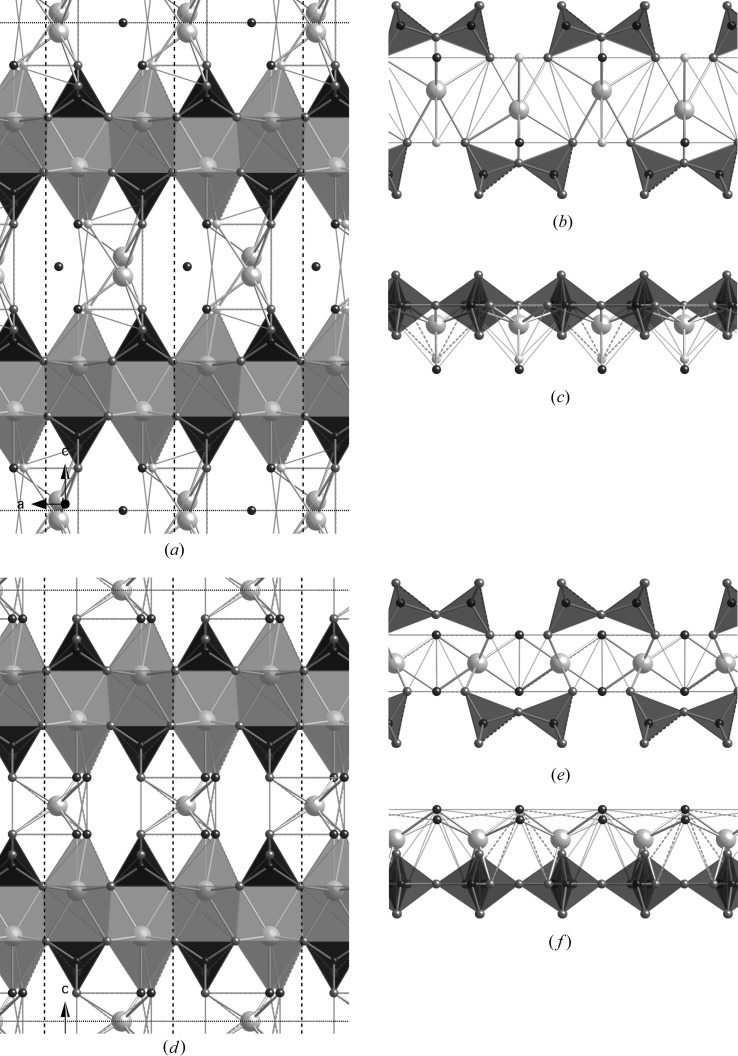
Two alternative hypothetical dimer structures derived from a staggered-chain orthotobermorite. In both cases, the space group is *B*11 *b* (No. 9, unique axis *c*, cell choice 1); *a* = 6.748, *b* = 7.3 Å, γ = 122.75°. (*a*)–(*c*) A version where the interlayer Ca atom is placed close to the vacant tetrahedral bridging site; in this version *c* = 21.5 Å; full details are in the CIF datablock T2_so_LS1. (*d*)–(*f*) A version where the interlayer Ca atom is offset from that position; in this version *c* = 19.0 Å; full details are in the CIF datablock T2_so_LS2. (*a*) and (*d*) are views along the **b** axis. The unit cell is indicated by black dotted lines. Just the silicate chains and interlayer Ca atoms are shown perpendicular to the **b** axis in (*b*) and (*e*) and along the **c** axis in (*c*) and (*f*).

The end-members of the dotted line on Fig. 5 ▸ [*i.e.* equation (16)] are a structure with infinite silicate chains and no interlayer Ca at Ca/Si = 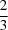, and an entirely dimeric structure at Ca/Si = 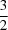, which will be referred to respectively as T∞ and T2 (after Richardson & Groves, 1992*a* ▸). It is therefore necessary to first develop models for these two end-member structures. All previous studies, including those discussed in §2, have concerned orthotobermorite structures, which as a consequence were used as a starting point in this work. Fig. 8 ▸ shows two hypothetical structures for a dimer that were established as having interlayer Ca atoms in the most plausible positions. The models were derived from an orthotobermorite structure that had staggered silicate chains (datablock T∞_11so) that was in turn developed from a double-chain structure that is given in Merlino *et al.* (2001 ▸). Figs. 8 ▸(*a*)–(*c*) are for a version where the interlayer Ca atom is placed close to the vacant tetrahedral bridging site (datablock T2_so_LS1) and Figs. 8 ▸(*d*)–(*f*) are for a version where the interlayer Ca atom is offset from that position (datablock T2_so_LS2). The faces of the interlayer Ca—O polyhedra are not shaded. Whilst the interlayer Ca atoms are coordinated to an appropriate number of O atoms, in both cases they are in distorted trigonal prism coordination – which is evident in Figs. 8 ▸(*c*) and (*f*) – rather than in octahedral coordination, which is commonly observed for calcium silicate hydrates. It is as a consequence not possible to adjust the *c* parameter (with *z*/*c* coordinates recalculated accordingly) to give values of average Ca—O distance and polyhedral volume for the interlayer Ca that are simultaneously consistent with the data in Fig. 7 ▸. Dimeric structures can also be generated easily from an orthotobermorite that has silicate chains where the bridging tetrahedra are adjacent to one another instead of staggered, but they suffer from the same Ca-coordination issue. The conclusion from these trial structures is that it is not possible to generate a dimeric model that is crystal-chemically consistent with known calcium silicate hydrates by using an orthotobermorite starting structure that has bridging tetrahedra that are adjacent to one another or that are staggered by *b*/2.

#### 5.2.2. Dimeric structures that are based on clinotobermorite   

**Figure 9:**
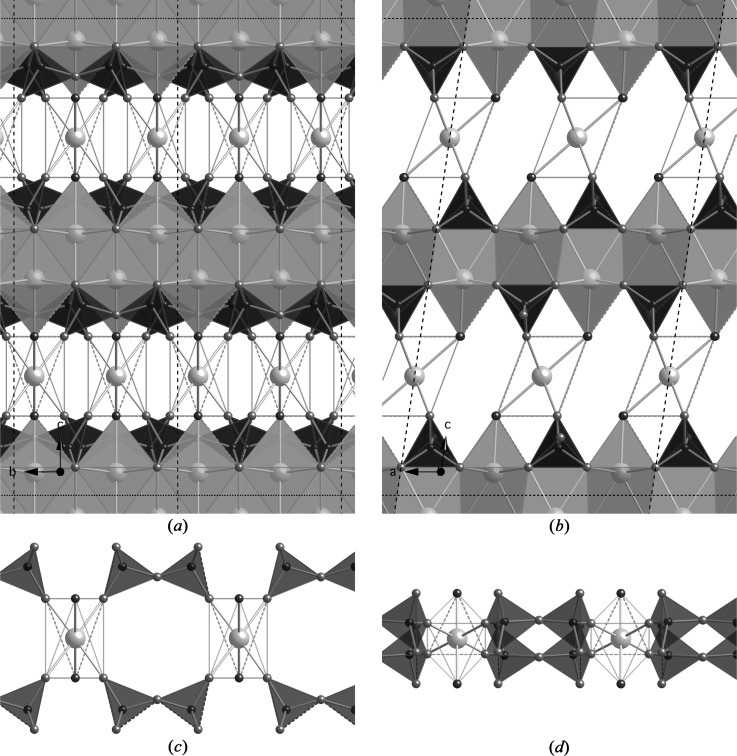
A hypothetical dimer derived from a clinotobermorite that had BT that were adjacent to one another. Space group *C*12/*c*1 (No. 15, unique axis *b*, cell choice 1); *a* = 11.35, *b* = 7.3, *c* = 21.5 Å, β = 98.4°; full details are in the CIF datablock T2_ac. (*a*) Viewed along the **a** axis (edge lengths are *b* and *c*sin β = 21.27 Å), and (*b*) viewed along the **b** axis (edge lengths *a* and *c*). The relationship of the interlayer Ca with the silicate tetrahedra is illustrated in (*c*) and (*d*).

The Ca-coordination problem that is encountered with the orthotobermorite models derives from the fact that the silicate chains that are clasped to successive Ca—O main layers are directly adjacent to one another (*i.e.* across the interlayer), as illustrated in Figs. 8 ▸(*a*) and (*d*). The problem is resolved by using clinotobermorite as the starting structure instead of an orthotobermorite. Again, starting models can be chosen that have bridging tetrahedra that are either adjacent to one another or that are staggered by *b*/2. The structure of a hypothetical dimer that is derived from a clinotobermorite structure that has bridging tetrahedral sites that are adjacent to one another is shown in Fig. 9 ▸(*a*) viewed along the **a** axis, and in Fig. 9 ▸(*b*) viewed along the **b** axis. The structure is monoclinic, space group *C*12/*c*1 (No. 15, unique axis *b*, cell choice 1); *a* = 11.35, *b* = 7.3, *c* = 21.5 Å, β = 98.4° (datablock T2_ac); it was developed from a double-chain structure given in Merlino *et al.* (2000 ▸). In contrast to the orthotobermorite, in this case the chains that are on successive main layers are not directly adjacent to one another, as shown in Fig. 9 ▸(*b*). This change in the position of the silicate chains results in the interlayer Ca atom being in octahedral coordination, which is evident in Fig. 9 ▸(*d*). The *c* parameter for this structure is set at a compromise value for the Ca—O distance and polyhedral volume: the average Ca—O distance for the interlayer Ca atom is slightly longer than the ideal value for sixfold coordinated Ca (2.43 Å rather than 2.40 Å) and the Ca—O polyhedral volume is slightly too small (16.57 Å^3^ compared with 17.35 Å^3^); nevertheless, inspection of Fig. 7 ▸ shows that both are plausible values. This model structure has the formula Ca_4_H_2_[Si_2_O_7_]_2_·Ca·4H_2_O, and so the Ca/Si = 1.25 and H_2_O/Si = 1.25; the calculated density is 2.302 g cm^−3^. It appears to be somewhat similar to Garbev, Bornefeld, Beuchle & Stemmermann’s (2008 ▸) dimer (*cf.* Fig. 9 ▸ *c* to the schematic illustration in their Fig. 8C), but without the water molecules that put their interlayer Ca in eightfold coordination, and it is also evident that their layer spacing of 11.7 Å, when compared with the value for this structure [*d*_002_ = 10.63 Å, *i.e.* (*c*/2)sin β] would result in an implausibly long average Ca—O distance.

**Figure 10:**
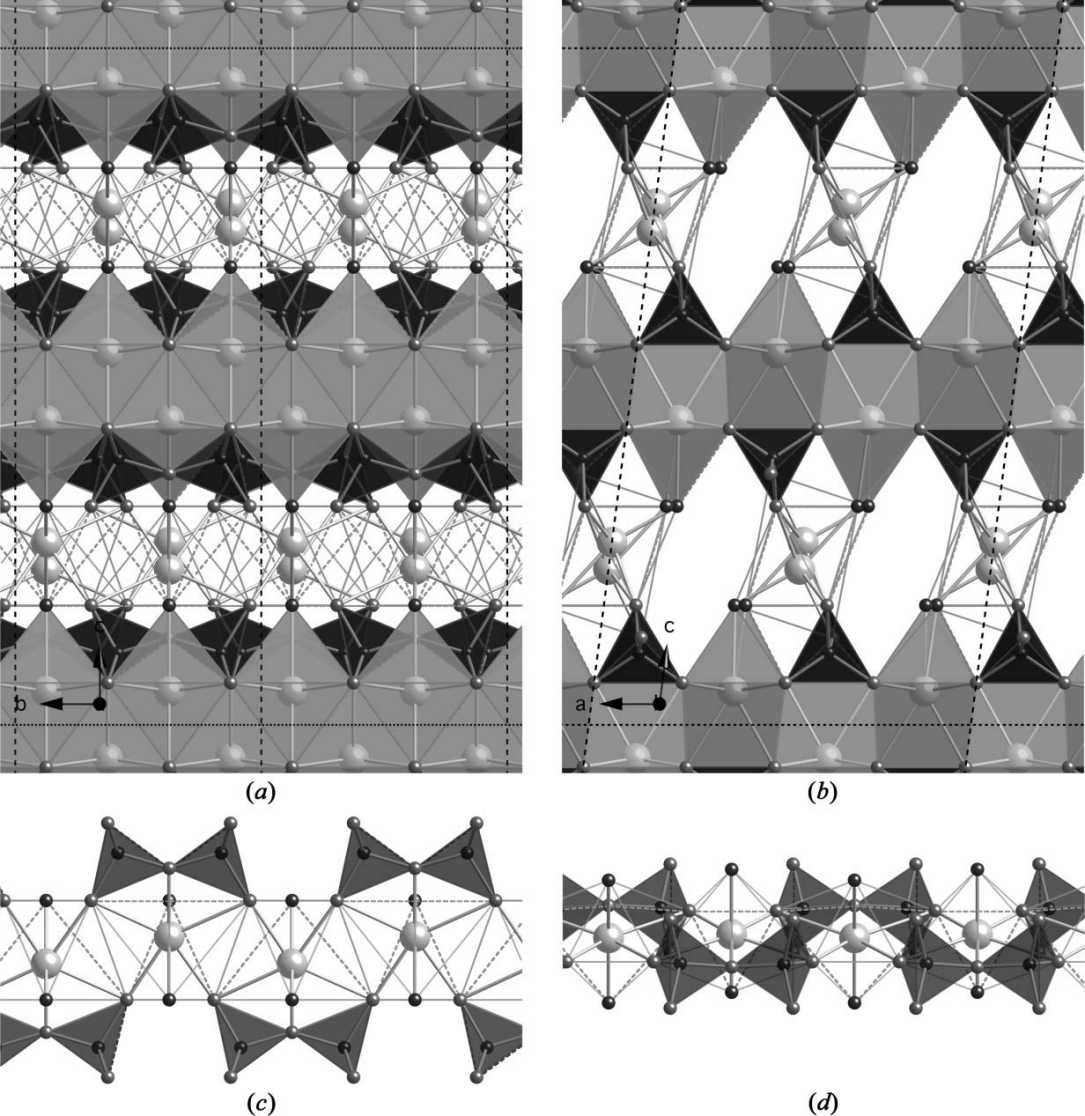
A hypothetical dimer derived from a staggered-chain clinotobermorite. Space group *P*12_1_/*c*1 (No. 14, unique axis *b*, cell choice 1); *a* = 11.35, *b* = 7.3, *c* = 20.25 Å, β = 97.28°; full details are in the CIF datablock T2_sc_LS1. (*a*) Viewed along the **a** axis (edge lengths are *b* and *c*sin β = 20.09 Å), and (*b*) viewed along the **b** axis (edge lengths *a* and *c*). The relationship of the interlayer Ca with the silicate tetrahedra is illustrated in (*c*) and (*d*).

**Figure 11:**
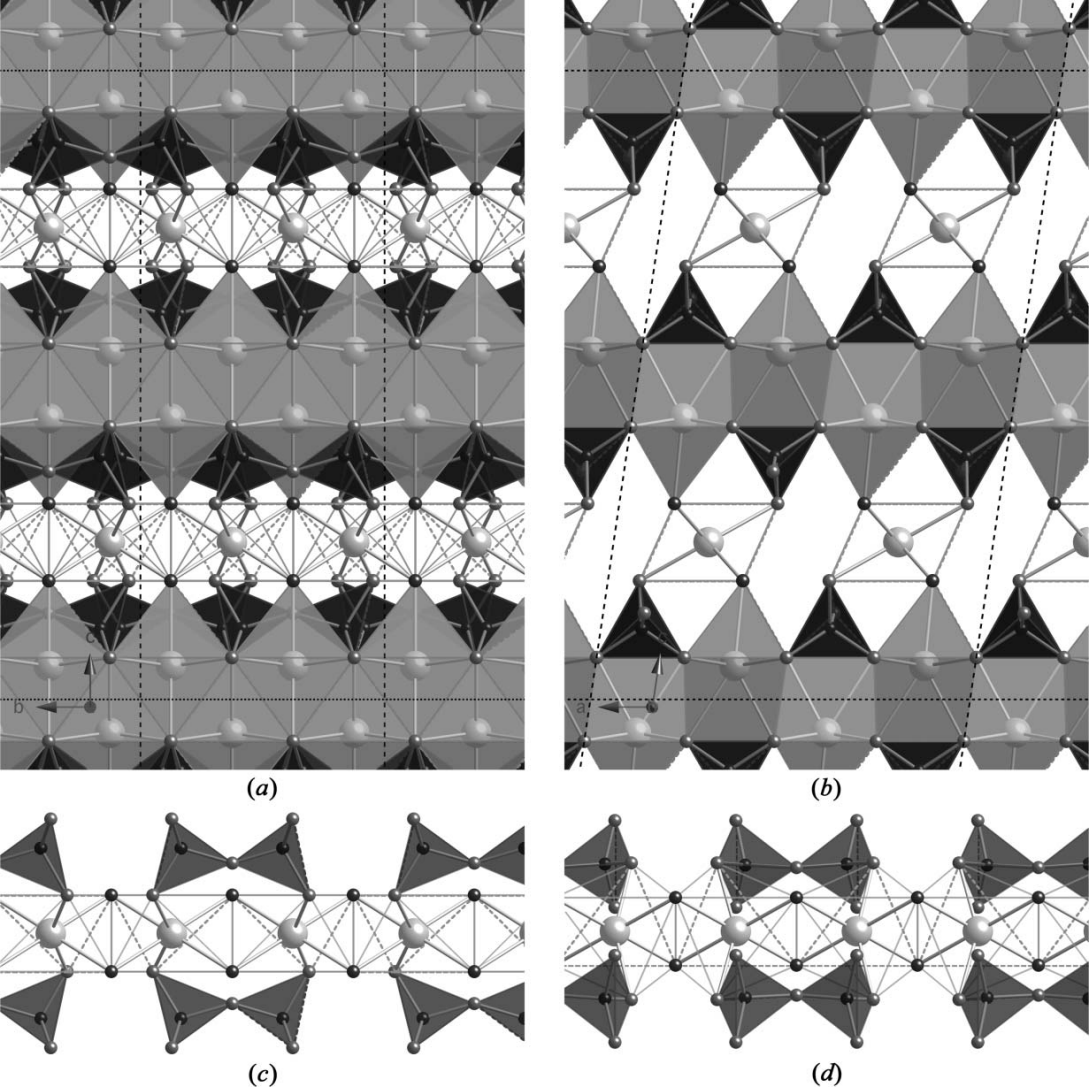
A hypothetical dimer derived from a staggered-chain clinotobermorite. Space group *P*12_1_/*c*1 (No. 14, unique axis *b*, cell choice 1); *a* = 11.35, *b* = 7.3, *c* = 19.0 Å, β = 98.4°; full details are in the CIF datablock T2_sc_LS2 (*a*) viewed along the **a** axis (edge lengths are *b* and *c*sin β = 18.796 Å), and (*b*) viewed along the **b** axis (edge lengths *a* and *c*).

A model dimer can also be derived from a clinotobermorite structure that had staggered silicate chains, and as with the orthotobermorite models, there are two alternative positions for the interlayer Ca atom that seem plausible. Figs. 10 ▸(*a*)–(*d*) illustrate a version where the interlayer Ca atom is placed close to the vacant tetrahedral bridging site, and Figs. 11 ▸(*a*)–(*d*) are for a version where the interlayer Ca atom is offset from that position. The first version (T2_sc_LS1) represents the situation where the same Ca atom charge compensates the negative charges of the chain-end O atoms that result from the omission of a single bridging tetrahedron, whereas the second version (T2_sc_LS2) involves two Ca atoms.

The T2_sc_LS1 structure is monoclinic, space group *P*12_1_/*c*1 (No. 14, unique axis *b*, cell choice 1); *a* = 11.35, *b* = 7.3, *c* = 20.25 Å, β = 97.28°. The structure is illustrated in Fig. 10 ▸(*a*) viewed along the **a** axis (the unit cell is indicated by black dotted lines and the edge lengths are *b* and *c*sin β = 20.09 Å), and Fig. 10 ▸(*b*) viewed along the **b** axis (edge lengths are *a* and *c*). The relationship of the interlayer Ca with the silicate tetrahedra is illustrated in (*c*) and (*d*). The formula is in this case Ca_4_[Si_2_O_7_]_2_·Ca_2_·4H_2_O and so Ca/Si = 1.5 and H_2_O/Si = 1.00. This model represents a more plausible version of the structure that is illustrated in Fig. 28 of Richardson (2004 ▸) for tobermorite-based dimeric C-S-H that had the minimum degree of protonation of the silicate chains [*w*/*n* = 0 in Richardson & Groves’ (1992*a* ▸) model]; Richardson’s (2004 ▸) figure was derived from an orthotobermorite structure. The layer spacing is 10.04 Å [*i.e.* (*c*/2)sin β] and the calculated density is 2.590 g cm^−3^. The interlayer Ca atom is coordinated to seven O atoms and the average Ca—O = 2.455 Å and the Ca—O polyhedral volume = 21.58 Å^3^, which are both consistent with the values from the regression analysis of the data for crystalline calcium silicate hydrates (Fig. 7 ▸), which are respectively 2.462 and 21.45 Å^3^.

Version T2_sc_LS2 is very similar to T2_sc_LS1, but with a different arrangement in the interlayer. The structure is illustrated in Fig. 11 ▸(*a*) viewed along the **a** axis and in (*b*) viewed along the **b** axis. The formula is again Ca_4_[Si_2_O_7_]_2_·Ca_2_·4H_2_O, Ca/Si = 1.5 and H_2_O/Si = 1.00. The *c* parameter has been reduced further to 19.0 Å, and so the layer spacing is 9.40 Å [*i.e.* (*c*/2)sin β]. The calculated density is 2.767 g cm^−3^. The interlayer Ca atom (Ca3) is coordinated to six O atoms and the average Ca3—O = 2.40 Å and the Ca3/3*A*—O polyhedral volume = 17.44 Å^3^, which are both close to the ideal values for Ca in sixfold coordination in crystalline calcium silicate hydrates (Fig. 7 ▸; ideal values are 2.40 Å and 17.35 Å^3^). These facts, together with the O—O distances in the structure, suggest strongly that the interlayer spacing for this structure must at 9.4 Å be close to the minimum that is crystal-chemically possible. It is relevant that this is essentially the same value as in the 9 Å tobermorites (9.3 Å; Merlino *et al.*, 1999 ▸, 2000 ▸).

**Figure 12:**
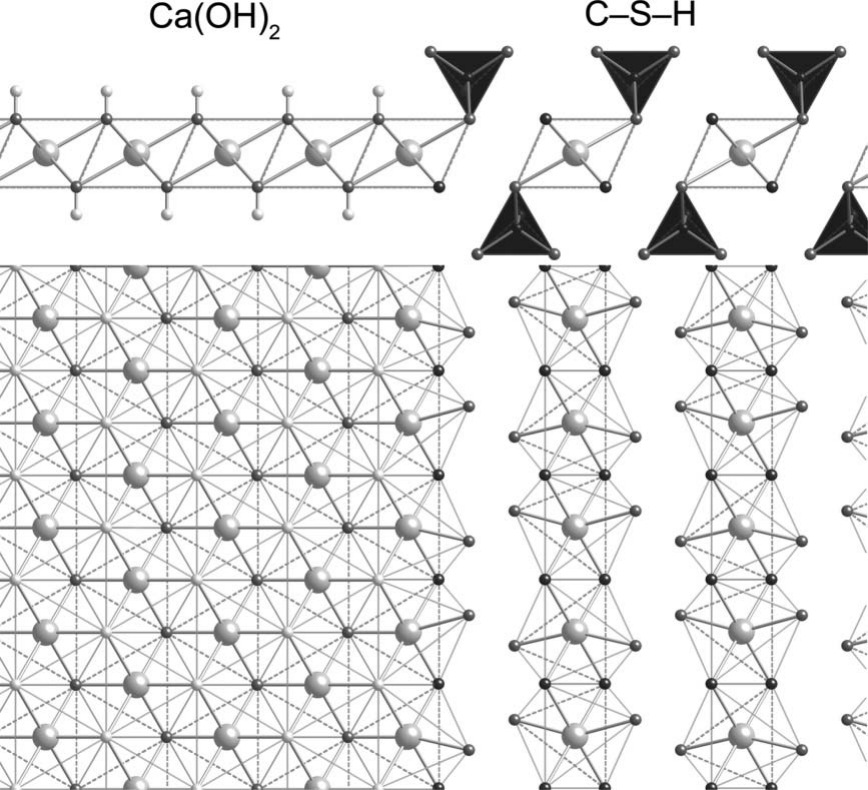
Schematic diagram that illustrates the possible topotactic relationship between the main layer of Ca(OH)_2_ and the interlayer of a hypothetical clinotobermorite-derived dimeric C-S-H(I).

The interlayer region in the T2_sc_LS2 structure consists of ribbons of Ca—O octahedra that run parallel to the **b** axis. These octahedra are very similar to those that are present in Ca(OH)_2_, although they are slightly distorted due to the need to respect the O—O distances of the silicate dimers that are clasped to either side. These ribbons of Ca—O octahedra and the associated silicate dimers are illustrated in Figs. 11 ▸(*c*) and (*d*): viewed along **a** in (*c*), with **c** up the page; and perpendicular to the layer in (*d*). The close similarity of these octahedra with those present in Ca(OH)_2_ offers a possible explanation for the intergrowth of C-S-H(I) and Ca(OH)_2_ that has been observed by TEM to occur in alkali-activated cements (Richardson & Groves, 1997 ▸; Richardson, 2004 ▸). This is illustrated in Fig. 12 ▸, which shows a possible topotactic relationship between the main layer of the Ca(OH)_2_ structure and the interlayer of this hypothetical dimeric C-S-H(I). This can be taken to represent the way that elements of Ca(OH)_2_-like structure are incorporated into C-S-H in the T/CH interpretation of Richardson & Groves’ (1992*a* ▸) model for C-S-H.

There is some compositional flexibility in this model structure. Full occupancy of the interlayer Ca site (Ca3) corresponds to *i* = 0 in formula (1). When the occupancy is reduced to 0.5, one half of the O1/O1*A*/O2/O2*A* sites carry a H atom, and the details change to: Ca/Si = 1.25; H_2_O/Si = 1.25; ρ = 2.605 g cm^−3^. This composition corresponds to *i* = 1 in formula (1). It should be noted that the occupancy of the Wat8/Wat9 sites is unaffected by a change in the occupancy of the interlayer Ca site because these water molecules are at the apices of main-layer Ca—O polyhedra. In fact the H_2_O/Si against Ca/Si data that are discussed in §6 indicate that at least for those particular data the occupancy of the Ca3 site is most likely to be 0.75, *i.e. i* = 0.5 in formula (1), in which case a quarter of the O1/O1*A*/O2/O2*A* sites carry a H atom and the details change to: Ca/Si = 1.375; H_2_O/Si = 1.125; ρ = 2.686 g cm^−3^.

The T2_sc_LS2 model structure with full occupancy of the interlayer Ca site is represented by a large unfilled, bold-outlined triangle in Fig. 1 ▸ at Ca/Si = 1.5. It is evident that it is in a reasonable position with regard to the experimental data (indeed, a slight increase in the value of *i* from 0 to 0.5 would move the point to the lower trend line). The same point is also represented in Fig. 5 ▸, where it is at the Ca-rich end of the dotted line that represents equation (16). The T2_sc_LS2 model is used in the rest of this paper to represent dimeric tobermorite-based structure because of its consistency with the experimental data on these figures and because of the possible explanation that it offers for the observed intergrowth of layers of C-S-H(I) and Ca(OH)_2_; it is therefore the structure that is referred to simply as ‘T2’. Nevertheless, this should not be taken to mean definitive exclusion by the author of the other models that seem to be crystal-chemically plausible; atomistic modelling studies might be expected to provide additional insight into the plausibility of the alternative models.

#### 5.2.3. Infinite-chain clinotobermorites: a hypothetical 14 Å clinotobermorite and a version that has no interlayer Ca   

A structure for the dimeric end-member of the dotted line in Fig. 5 ▸ [equation (16)] that is crystal-chemically plausible has been developed in §5.2.2. The next structure that is needed is the other end-member, *i.e.* a structure that has infinite silicate chains and no interlayer Ca (Ca/Si = 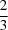), which will be referred to as T∞ (after Richardson & Groves, 1992*a* ▸). Since it was only possible to develop a dimeric model successfully that is based on a clinotobermorite, it is most likely that the infinite-chain end member is also based on clinotobermorite, and since the best model was derived from a staggered-chain structure (*i.e.* T2_sc_LS2), it would seem to be sensible to use the same arrangement.

14 Å clinotobermorite has not been observed in nature (regardless of the relative position of adjacent silicate chains), but by analogy with the orthotobermorites it seems possible that it could occur, and from §5.2.2 it would appear to be relevant to models for the structure of C-S-H(I). The development of this model commenced with the production of a slightly idealized version of the monoclinic MDO_1_ polytype of 11 Å clinotobermorite that is given in Table 4 of Merlino *et al.* (2000 ▸), which is a double-chain structure. Merlino *et al.*’s 11 Å structure is in space group *Cc*; idealization of the structure allowed an increase in symmetry to space group *C*2/*c* (*a* = 11.35, *b* = 7.3, *c* = 22.68 Å, β = 97.28°; full details are given in the CIF datablock T∞_11ac). Whilst the minimum Ca—O distance for the interlayer Ca atom in the 11 Å model is rather short (2.203 Å), it is not shorter than the minimum distance that has been observed by experiment in calcium silicate hydrates in general (§5.1) and it is in fact very similar to the minimum in Merlino *et al.*’s structure (2.236 Å). In addition, the maximum in the model is somewhat shorter than in Merlino *et al.*’s structure (2.862 Å compared with 2.957 Å), which is more consistent with the data in §5.1. The powder XRD patterns calculated from the model structure and Merlino *et al.*’s are closely similar if the lattice parameters are adjusted to be the same. In the next stage, the layer spacing was increased from 11 Å to around 14 Å and the central Ca—O slab preserved by recalculation of the *z*/*c* coordinate of the atoms in the asymmetric unit. In addition, it was necessary to move the interlayer Ca atom and a water molecule. The space group is *C*2/*c*; *a* = 11.35, *b* = 7.3, *c* = 28.8 Å, β = 95.5°; full details are given in the CIF datablock T∞_14ac. Finally, the silicate chains were staggered with a shift of *b*/2, with the displacement of the chains resulting in a reduction in symmetry to space group *P*2_1_/*c*; *a* = 11.35, *b* = 7.3, *c* = 28.8 Å, β = 95.5°; T∞_14sc. The structural formula is Ca_5_(Si_6_O_16_(OH)_2_)(H_2_O)_7_, which corresponds to *i* = 1 in formula (1) but it is possible to vary the contents of the interlayer. Selected bond distances for the structure are given in Table 3 ▸. The average Ca—O distance for the interlayer Ca (*i.e.* Ca3) is 2.39 Å, which is close to the value that would be expected for Ca in sixfold coordination; indeed, the maximum, average and minimum Ca—O and Si—O distances are all consistent with the values that are calculated from known structures of calcium silicate hydrates (Fig. 7 ▸). In this case it would seem not to be possible to have *i* < 1 (*i.e.* the occupancy of the interlayer Ca site > 0.5) because the Wat12 and Wat14 sites would both have to be occupied, but they are too close to one another. In principle, a lower value could be accommodated by replacing those molecules by a single water molecule at (0.75, 0.125/0.625, 0.25) that would coordinate to adjacent Ca ions with the long Ca—O distance of 2.84 Å; however, that would result in a Ca—O polyhedral volume that is much too large for sixfold coordination (20.98 Å^3^) and so is unlikely.

The infinite-chain end member (T∞) of equation (16) has a Ca/Si ratio of 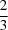, *i.e. i* = 2. The Ca/Si ratio is reduced easily from 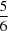 to 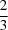 by setting the occupancy of the interlayer Ca site to 0. Since the value of the layer spacing is in part determined by the coordination requirements of the interlayer Ca atom, it is likely to reduce if that site is unoccupied. The size of the reduction is then likely to be dependent upon the number of water molecules that are retained in the interlayer, which is dependent upon the extent of drying and so will be variable. Complete removal of molecular water would result in a H_2_O/Si ratio of just 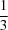 (*i.e.* Si—OH/Si = 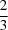), which is the value established by experiment for the most highly dried samples of C-S-H(I) (see Fig. 2 ▸). Fig. 8 ▸(B) in Garbev, Bornefeld, Beuchle & Stemmermann (2008 ▸) is a schematic illustration of a structural arrangement for a single-chain orthotobermorite with Ca/Si = 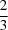, composition Ca_4_H_4_[Si_3_O_9_]_2_·4H_2_O. The structure consists of tobermorite slabs that have staggered silicate chains, no interlayer Ca atoms, and a layer spacing of 13.3 Å. An equivalent structure is derived easily from T∞_14sc that has the same composition and layer spacing as Garbev, Bornefeld, Beuchle & Stemmermann’s (2008 ▸) model (space group *P*2_1_/*c*; *a* = 11.35, *b* = 7.3, *c* = 26.715 Å, β = 95.5°; full details in T∞_sc_noCa_LS1); the calculated density is 2.089 g cm^−3^. A larger layer spacing is preferred for this work (14.15 Å) so that the developed models are consistent with a wider range of experimental data. This higher value can be achieved whilst still maintaining reasonable O—O distances by using a slightly different arrangement of water molecules in the interlayer (the *z*/*c* coordinates are also recalculated). The space group is *P*2_1_/*c*; *a* = 11.35, *b* = 7.3, *c* = 28.43 Å, β = 95.5°; details in T∞_sc_noCa_LS2.

The clinotobermorite-based models derived so far are for dimeric and infinite-chain silicate structures, which have SOF_BT_ values of 0 and 1, respectively. The most straightforward explanation for the trend on the plot of SOF_BT_ against Ca/Si ratio [*i.e.* the dotted line in Fig. 5 ▸ that represents equation (16)] is that a mix of these two structure types could account for C-S-H preparations that have intermediate fractions of vacant bridging sites: a 1:1 mix of dimeric (T2) and infinite-chain (T∞) structure would give the same fraction of vacant bridging sites as the pentamer (*i.e.* SOF_BT_ = 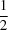); a 1:2 mix would give the same fraction as the octamer (SOF_BT_ = 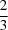); a 1:3 mix the same as the undecamer (SOF_BT_ = 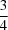) *etc*. This combination of T2 and T∞ layers also provides a plausible explanation for the presence of dimeric structure down to low Ca/Si ratios, as observed by Brunet *et al.* (2004 ▸). However, it must be emphasized that vacant bridging sites must be present in the T∞ layer in some real preparations because of the observed Q^1^–Q^2P^ correlation as well as Q^1^–Q^1^.

#### 5.2.4. Structural models for ‘pentamer’, ‘trimer’ and ‘undecamer’   

**Figure 13:**
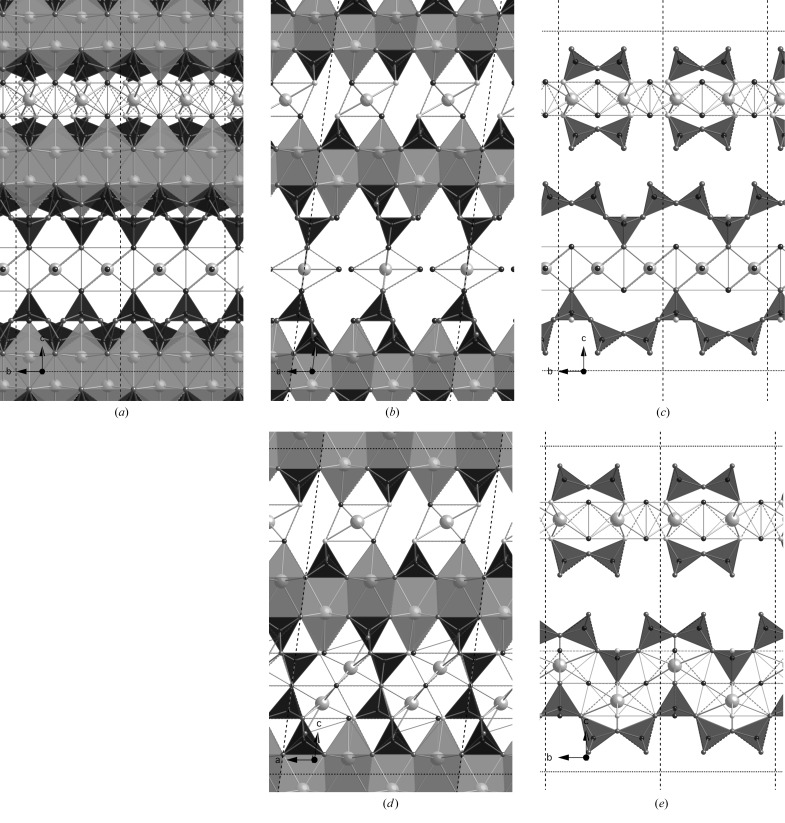
A hypothetical ‘pentamer’ derived from a staggered-chain clinotobermorite, (*a*) viewed along the **a** axis (edge lengths are *b* and *c*sin β = 23.790 Å), and (*b*) viewed along the **b** axis (edge lengths *a* and *c*). The space group is *C*1; *a* = 11.35, *b* = 7.3, *c* = 23.9 Å, α = 90, β = 95.5, γ = 90°; full details in the CIF datablock T5_14sc. Single silicate chains and associated interlayer Ca atoms are shown in (*c*) viewed along the **a** axis. (*d*) The same as (*b*) but for a ‘pentamer’ where the T∞ part is derived from 11 Å clinotobermorite rather than 14 Å; space group *C*1; *a* = 11.35, *b* = 7.3, *c* = 20.84 Å, α = 90, β = 97.28, γ = 90°; full details in the CIF datablock T5_11sc; edge lengths *b* and *c* sin β = 20.67 Å. The single silicate chains and associated interlayer Ca atoms are shown in (*e*) viewed along the **a** axis.

A hypothetical ‘pentamer’ that is derived from a clinotobermorite structure that has staggered silicate chains is shown in Fig. 13 ▸: it is viewed in (*a*) along the **a** axis and in (*b*) along the **b** axis. Single silicate chains and associated interlayer Ca atoms are shown in (*c*). Although this ‘pentamer’ model structure – that will be referred to as ‘T5’ – still has the monoclinic cell of the T2 and T∞ models from which it is derived, the rotational symmetry has been lost and so the resulting structure is triclinic, space group *C*1 (No. 1, setting 2); *a* = 11.35, *b* = 7.3, *c* = 23.9 Å, α = 90, β = 95.5, γ = 90°, *Z* = 4; full details in T5_14sc. The Ca/Si ratio is 1.0, which is consistent with equation (16). The site occupancy factor for the interlayer Ca sites in both parts of the structure = 1-SOF_BT_ = 0.5. For the T2 part, the fraction of O atoms that are at the end of the silicate dimers that carry a H atom = 1-SOF_BT_ = 0.5 (*i.e.* the OH1 atoms) and for the T∞ part the fraction of O atoms that are at the apex of the bridging tetrahedra that carry a H atom = 1-(2 × SOF_BT_) = 0 (*i.e.* atoms O3_4 and O3_9). In terms of formula (1), the formula is Ca_4_[Si_5_□_1_O_16_]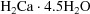, *i.e. i* = 2 × SOF_BT_ = 1. The H_2_O/Si ratio is 1.10 and the calculated density is 2.291 g cm^−3^. Most of the interatomic distances are as reported in Table 3 ▸ for the model 14 Å clinotobermorite. The average Ca—O distances and polyhedral volumes for the interlayer Ca atoms (Ca2 and Ca3) are consistent with the values that are expected for sixfold coordinated Ca in calcium silicate hydrates, *i.e.* with the regression analysis equations in Fig. 7 ▸ (average Ca3—O = 2.40 Å; Ca3—O polyhedral volume = 17.48 Å^3^; average Ca2—O = 2.39 Å; Ca2—O polyhedral volume = 17.82 Å^3^). The layer spacing *d*_002_ = 11.895 Å [*i.e.* (*c*/2)sin β], which is represented by a large unfilled, bold-outlined triangle on Figs. 1 ▸ and 5 ▸ at Ca/Si = 1.0. As with the points for the T2 and T∞ models, it is evident that the T5 point is in a reasonable position with regard to the experimental data.

The plot of layer spacing, *d*_00*l*_, against Ca/Si ratio (Fig. 1 ▸) indicates that the most suitable infinite-chain model is the one that is derived from 14 Å clinotobermorite rather than from an 11 Å structure. Nevertheless, a model structure that has the T∞ part based on 11 Å clinotobermorite is of interest because it would represent a higher degree of drying. 11 Å clinotobermorite that has staggered silicate chains has not been observed in nature nor synthesized and so there is no structure available. However, such a model is straightforward to create from the cross-linked structure that is described in §5.2.3 (*i.e.* T∞_11ac) by a shift of *b*/2 in the silicate chains (see T∞_11sc). A ‘pentamer’ model structure that uses this 11 Å clinotobermorite model as the T∞ module is illustrated in Figs. 13 ▸(*d*) and (*e*), which should be compared with Figs. 13 ▸(*b*) and (*c*), respectively; full details are given in the CIF datablock T5_11sc (space group *C*1; *a* = 11.35, *b* = 7.3, *c* = 20.84 Å, β = 97.3°). This change results in a decrease in the *d*_002_ spacing from 11.89 to 10.34 Å and in the H_2_O/Si ratio from 1.10 to 0.90 (the Ca/Si ratio is unchanged).

**Figure 14:**
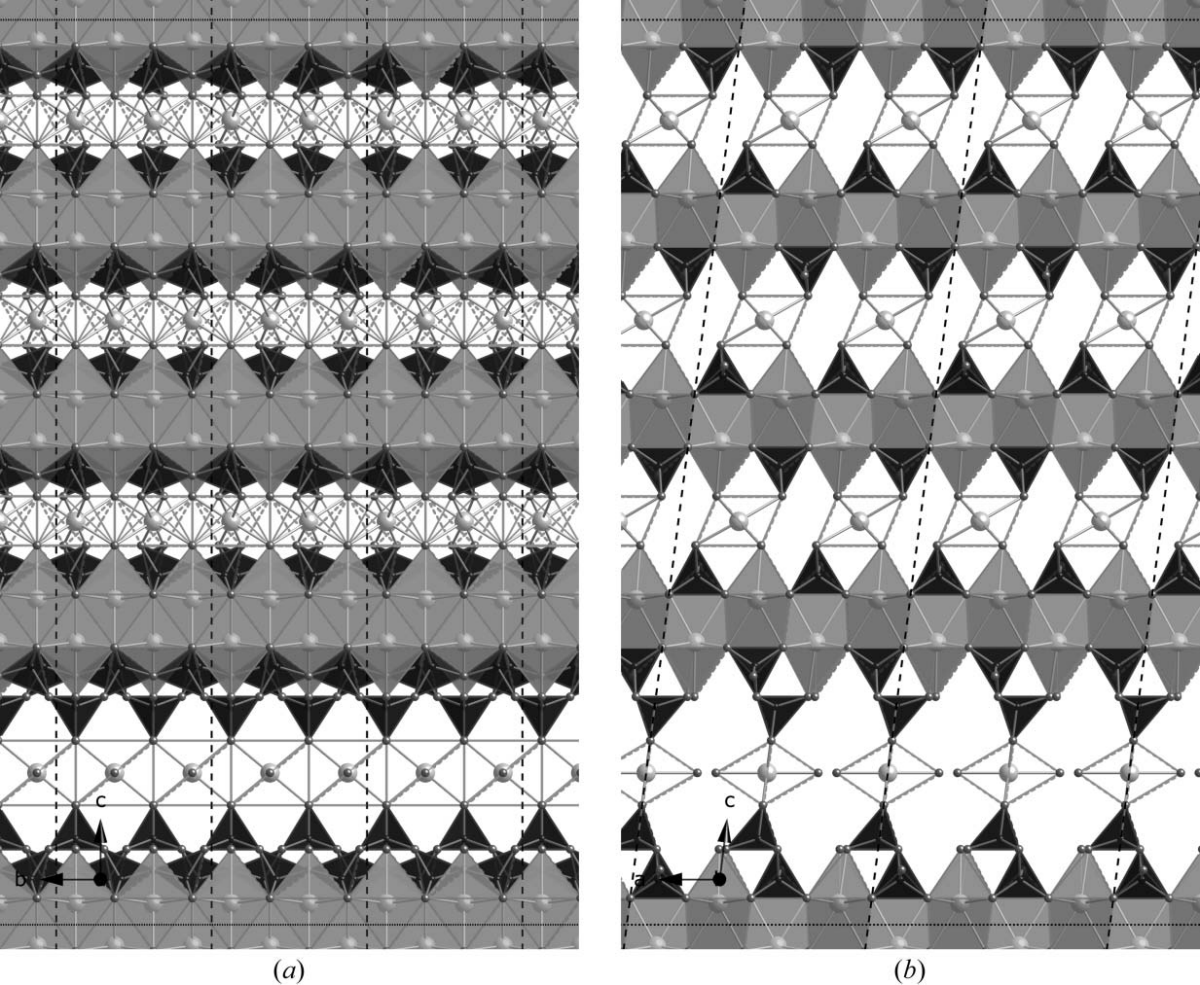
A hypothetical ‘trimer’ derived from a staggered-chain clinotobermorite (*a*) viewed along the **a** axis (edge lengths *b* and *c*sin β = 42.702 Å), and (*b*) viewed along the **b** axis (edge lengths *a* and *c*). The formula is Ca_11_(Si_9_O_28_(OH)_2_)(H_2_O)_8.5_; the calculated density is 2.494 g cm^−3^; *i* = 2 × SOF_BT_ = 0.5. The space group is *C*1; *a* = 11.35, *b* = 7.3, *c* = 42.9 Å, α = 90, β = 95.5, γ = 90°; *Z* = 4; full details are in the CIF datablock T3_14sc.

The T2 and T∞ layers can be combined in ratios other than 1:1: as examples, 3:1 produces a structure that has only a quarter of the tetrahedral bridging sites occupied (*i.e.* SOF_BT_ = 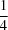; *v* = 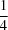) and so the average length of the silicate chains is 3 (it is important to note that this is an average rather than absolute chain length); whilst 1:3 results in three quarters of the tetrahedral bridging sites occupied (SOF_BT_ = 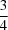, *v* = 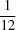) and the average length of the silicate chains is 11. Model structures for T3 and T11 are illustrated in Figs. 14 ▸ and 15 ▸. In both cases, the space group is *C*1 (No. 1, setting 2); *a* = 11.35, *b* = 7.3 Å, α = 90, β = 95.5, γ = 90°, *Z* = 4. For T3, *c* = 42.9 Å, and for T11, *c* = 52.7 Å; full details are in the CIF datablocks T3_14sc and T11_14sc. These models are also represented by large unfilled, bold-outlined triangles on Figs. 1 ▸ and 5 ▸, and it is again evident that the points are in a reasonable position with regard to the experimental data.

## 6. Calculated powder XRD patterns, density and water content for the model structures   

**Figure 16:**
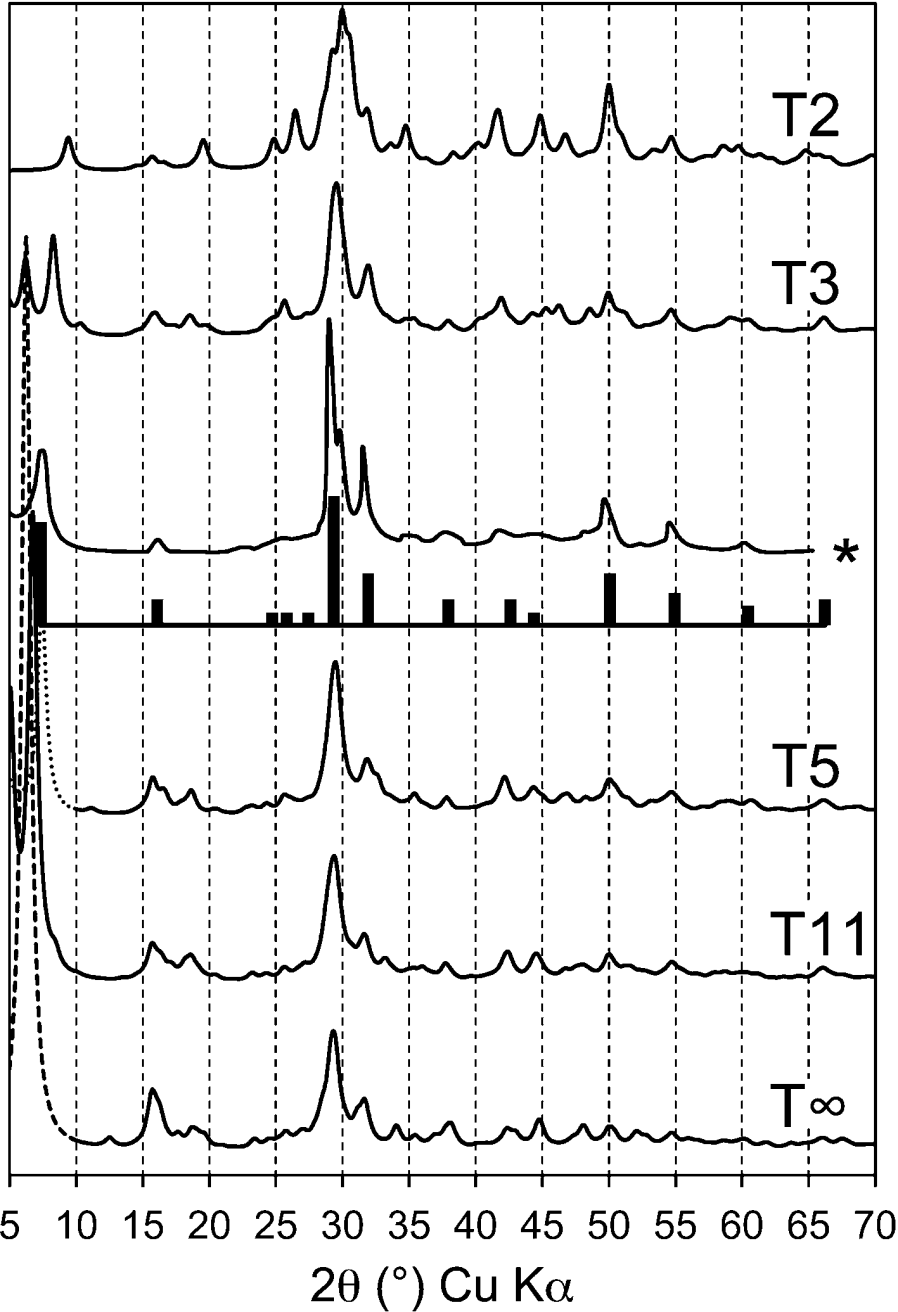
Calculated powder XRD patterns for the model C-S-H(I) structures developed in this paper (monochromatic Cu *K*α radiation, λ = 1.5406 Å). The patterns were calculated using *CrystalDiffract*® (CrystalMaker Software Ltd, 2011 ▸) assuming isotropic crystals of size 10 nm. The inset bar-pattern represents typical experimental data, as discussed in §2. An experimental pattern is also included, indicated by *. It was extracted from Fig. 3 ▸ of Cong & Kirkpatrick (1996 ▸; sample SCFUMd).

It has been demonstrated that the main layer-spacing peak for each of the model structures is consistent with the linear decrease that is observed to occur with increasing Ca/Si ratio (Fig. 1 ▸). Calculated powder XRD patterns for the model structures are shown in Fig. 16 ▸ (monochromatic Cu *K*α radiation, λ = 1.5406 Å). The patterns were calculated using *CrystalDiffract* ^®^ (CrystalMaker Software Ltd, 2011 ▸) assuming isotropic crystals of size 10 nm, although it is important to note that the crystals in real samples are likely to be anisotropic, which would affect the relative intensities and widths of the peaks (Renaudin, Russias, Leroux, Frizon & Cau-dit-Coumes, 2009 ▸). Experimental data for C-S-H(I) are inset in Fig. 16 ▸, including a pattern that was extracted from Fig. 3 ▸ of Cong & Kirkpatrick (1996 ▸; sample SCFUMd, indicated by *) and as bars that take into account the points that are listed in §2; the bar that represents the basal reflection is set at 12 Å to facilitate comparison with the pattern calculated for the T5 model. It is evident that the main features of the experimental data are present in the calculated patterns. More accurate simulations of powder XRD patterns – or the use of the models in Rietveld refinements – would need to take account of the experimental value for SOF_BT_ and the Ca/Si ratio of the C-S-H should be determined unambiguously by direct measurement, *e.g.* by TEM-EDX of dispersed samples. It is noted in §5.2.3 that in real C-S-H(I) preparations some BT must be missing from the T∞ part of the structures because of the experimental observation of Q^1^–Q^2P^ correlation. It is likely that other types of defects are also present, such as stacking faults, which will affect the XRD pattern.

The calculated density of the model structures increases linearly with Ca/Si ratio; the (slightly idealized) relationship is: *D*_c_ (g cm^−3^) = Ca/Si + 1.25.

It was noted in §2 that the H_2_O/Si ratio of C-S-H(I) preparations increases with increasing Ca/Si and that the value depends on the severity of the drying, Fig. 2 ▸. The H_2_O/Si ratio of the most highly dried samples (*i.e.* those in the lower part of Fig. 2 ▸) increases essentially linearly with an increase in the Ca/Si ratio. The lowest value is consistent with a totally collapsed tobermorite-like phase, *i.e.* essentially a fully protonated 9 Å single-chain tobermorite, formula Ca_4_Si_6_O_14_(OH)_4_ [*i.e.* *i* = 2, *v* = 0, *f* = 0, *m* = 0 in formula (1)] and so H_2_O/Si = 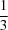 and Ca/Si = 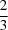. The observed increase in H_2_O/Si ratio with increasing Ca/Si ratio must involve the presence of vacant tetrahedral sites, a reduced number of hydroxyl groups and the introduction of interlayer Ca. The trend in Fig. 2 ▸ for the most highly dried samples indicates that any dimeric structure present at higher Ca/Si ratio must have slightly less than the maximum amount of interlayer Ca that is theoretically possible, in fact for purely dimeric structure, *i* = 0.5 rather than *i* = 0. The structural-chemical formula for the most highly dried C-S-H(I) (*i.e.* without destroying the tobermorite-like structure) is therefore as given in formula (20), with *i* = 2–4.5*v* [*i.e. i* = 2 for infinite chains (*v* = 0) and *i* = 0.5 for dimeric chains (*v* = 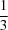)].
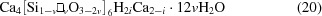
The Ca/Si ratio is
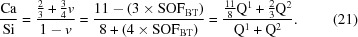
Also the H_2_O/Si ratio is
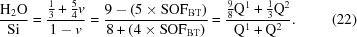
Combining equations (21) and (22) gives
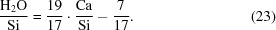
Equation (23) is represented by the lower thick line on Fig. 2 ▸. It is evident that this equation satisfactorily represents the data for the more highly dried preparations. The upper black line represents the same as the lower line but with one additional water molecule per Si atom. The bold unfilled diamonds represent the model structures that are developed in this paper and the five grey diamonds represent the positions for the least hydrated versions of them (from left to right, the T∞, T11, T5, T3 and T2 structures). It is evident that the positions of the diamonds are consistent with the data; much of the scatter in the data can as a consequence be interpreted as corresponding to different degrees of drying. The long-dashed lines correspond to an intermixture of T2 (*i* = 0.5) with CH.

The cross symbols are for C-S-H(II) (Gard & Taylor, 1976 ▸) with different degrees of drying; the positions are consistent with the possibility that some of the data points at higher Ca/Si ratio include C-S-H(II), which would be consistent with T/J models for the structure of C-S-H in hardened cement pastes (Taylor, 1986 ▸; Richardson & Groves, 1992*a* ▸). The crossed-square symbol represents Brunauer *et al.*’s (1958 ▸) bottle-hydrated C_3_S sample that was almost fully reacted, which is almost coincident with the point for the T2 (*i* = 0) model developed here. The measured density for their sample is also consistent with the value calculated for the T2 (*i* = 0) model.

**Figure 17:**
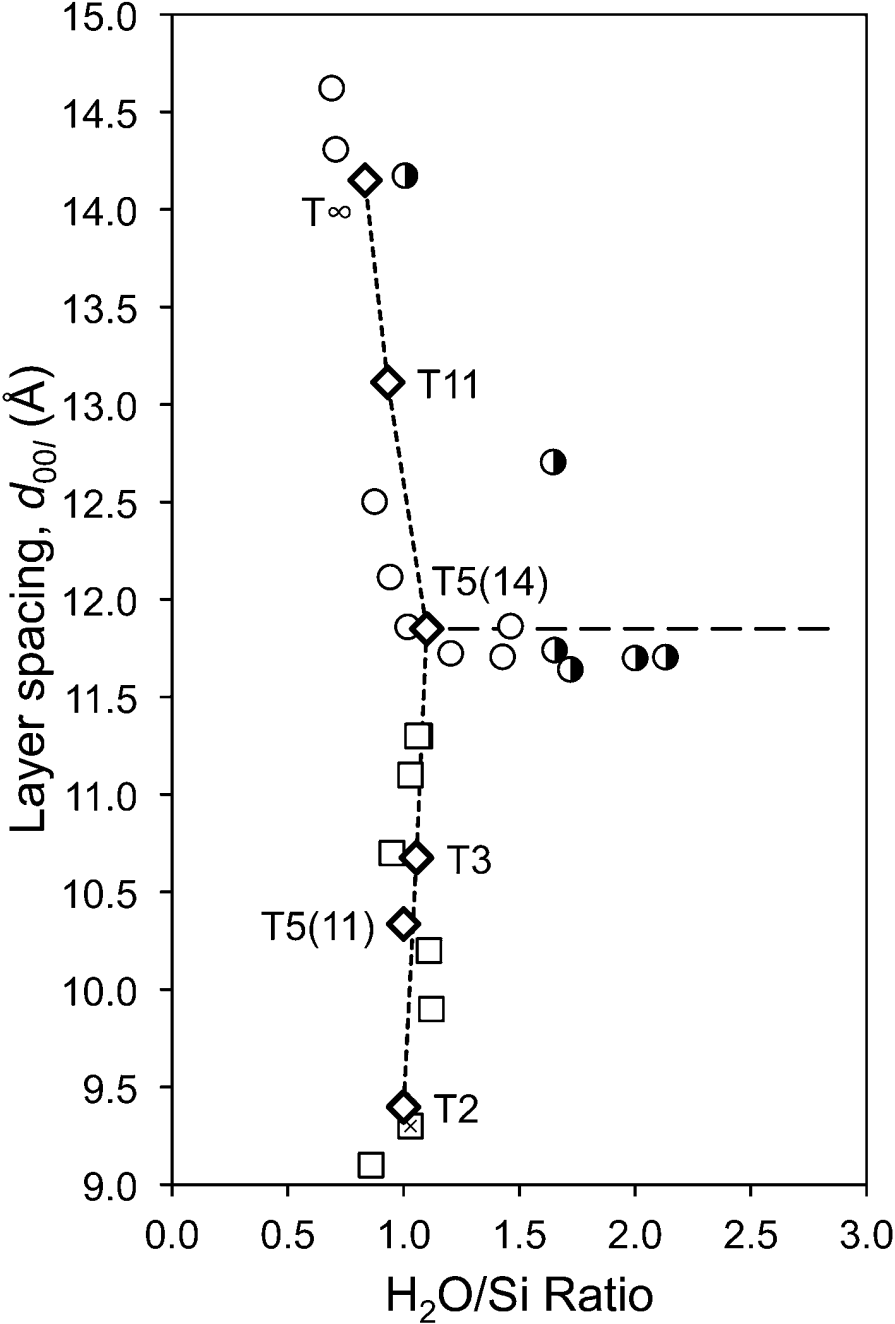
Plot of the layer spacing against the H_2_O/Si for C-S-H(I) preparations. The data are from: Brunauer *et al.* (1958 ▸) (crossed square); Cong & Kirkpatrick (1996 ▸) (unfilled circles: SCFUM series dried at 383 K); Renaudin, Russias, Leroux, Frizon & Cau-dit-Coumes (2009 ▸) (half-filled circle); Taylor & Howison (1956 ▸) (unfilled squares: Group B samples). The bold unfilled diamonds represent the model structures that are developed in this paper.

Fig. 17 ▸ is a plot of the layer spacing against the H_2_O/Si ratio for C-S-H(I) preparations. The sources of the data are given in the figure caption. The points for the 14 Å clinotobermorite-derived model structures are joined by the short-dash line. It is evident that the models are consistent with the observed variation in the layer spacing and H_2_O/Si ratio. The figure also supports the view given earlier that many C-S-H(I) preparations are in fact mixtures of C-S-H with a second phase; in this case the long-dash line indicates intermixture with T5(14).

## 7. The substitution of Si by Al   

**Figure 18:**
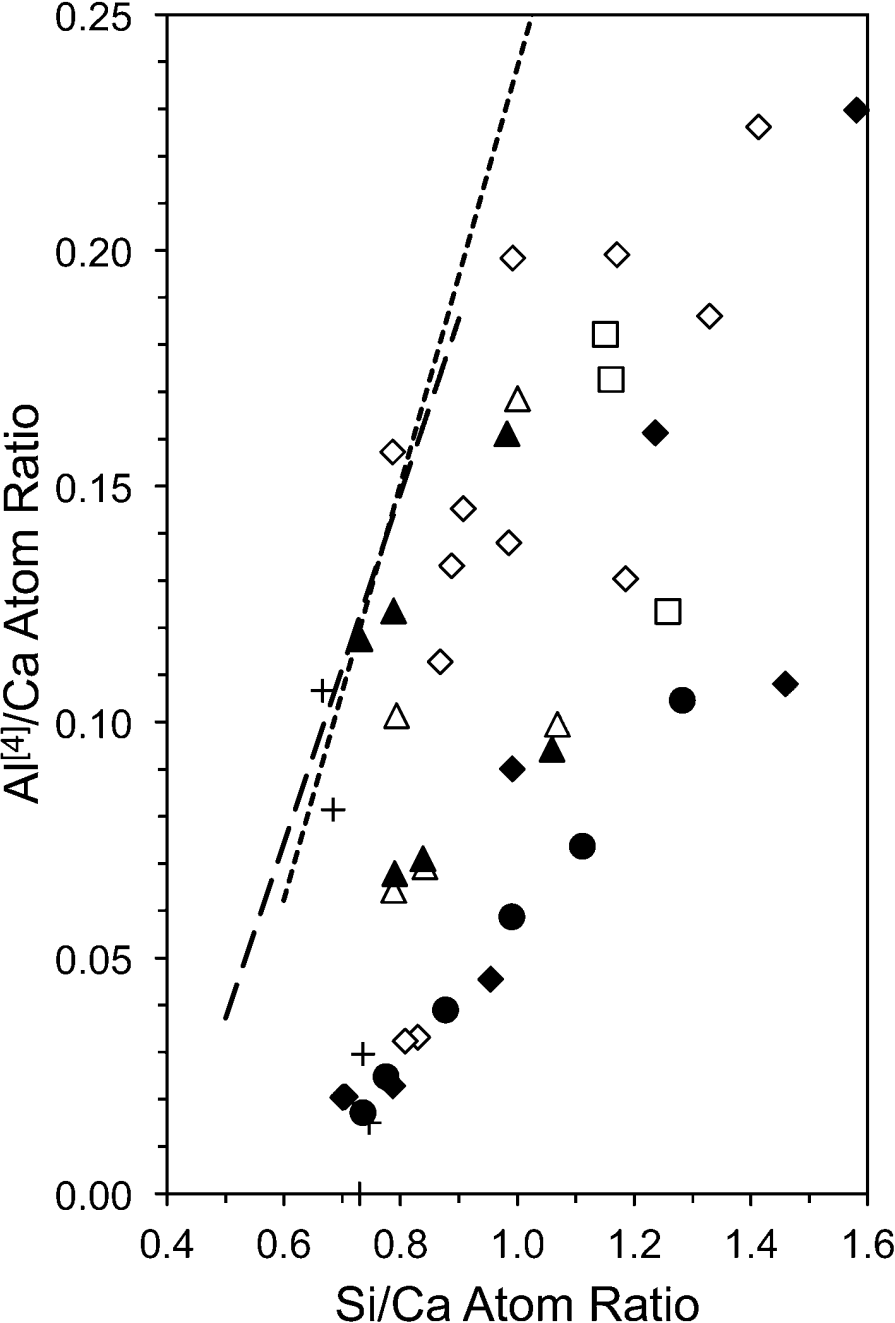
Plot of Al/Ca against Si/Ca ratio for C-A-S-H(I) preparations. The data are from Faucon, Petit *et al.* (1999 ▸) (unfilled diamond); Faucon, Delagrave *et al.* (1999 ▸) (filled diamond); Sun *et al.* (2006 ▸) (unfilled and filled triangles represent data at 1 and 4 weeks, respectively); Renaudin, Russias, Leroux, Cau-dit-Coumes & Frizon (2009 ▸) (filled circle), Pardal *et al.* (2012 ▸) (unfilled square). Al^[5]^ and Al^[6]^ are not included. The dashed lines represent the C-A-S-H that forms in real cements: the data are for neat Portland cement pastes and blends of Portland cement with blast-furnace slag, pulverized fuel ash and metakaolin; they are from Girão (2007 ▸); Girão, Richardson & Brydson (2007 ▸); Girão, Richardson, Porteneuve & Brydson (2007 ▸); Girão *et al.* (2010 ▸); Love *et al.* (2007 ▸); Richardson & Groves (1992*b* ▸, 1993*b* ▸, 1997 ▸); Taylor *et al.* (2010 ▸).

Aluminium is included in the structural-chemical formulae that are given in §3 because it is the main substituent for Si in C-S-H phases. Fig. 18 ▸ is a plot of the Al/Ca against Si/Ca ratio for synthetic C-A-S-H(I) preparations. The level of substitution that occurs in the C-A-S-H(I) that is present in alkali hydroxide-activated cement pastes is indicated by the short-dash line that extends to higher Al/Ca and Si/Ca ratios; it is the regression analysis equation from TEM-EDX analyses. The longer-dash line is for the water-activated pastes (*i.e.* that contain C-A-S-H that gives a poor diffraction pattern). The linear relationship between Al/Ca and Si/Ca ratios is essentially the same regardless of the type of activation (and so crystallinity of the C-A-S-H) or type of cement. The sources of all the data are given in the figure caption. It is evident that the points for the synthetic preparations mostly fall to the Si-rich side of the lines that represent the C-A-S-H that forms in real cements, with a few data points falling essentially on the lines. The data for the latter points can therefore be taken to best reflect the material that forms in real cements. The fact that most of the data for the synthetic preparations fall to the right of the lines suggests that the amount of Al that is incorporated into the C-A-S-H in real cements represents a practical maximum and that the synthetic preparations of most studies cannot be taken to represent the C-A-S-H that forms in real cements.

It was noted in §3 that Al substitutes for Si only at bridging sites and that the site occupancy factors for Si and Al at the bridging site can be calculated using equations (11) and (12). The models developed in §5 can therefore be extended easily to incorporate Al. Since occupied bridging sites only occur in the T∞ modules of the model structures, Al that is in fourfold coordination must be restricted to those parts of the structures. Consideration of Taylor *et al.*’s (2010 ▸) data for C-A-S-H that is present in 20-year-old water-activated blast-furnace slag/Portland cement blends indicates that *f* = 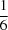 in every T∞ module in all of the model structures and so it would seem plausible that the tetrahedral bridging sites are occupied alternately by Al and Si atoms. The substitution of Al^3+^ for Si^4+^ necessitates the presence of additional interlayer ions to maintain charge balance, either monovalent alkali or Ca^2+^ cations, which are represented by the contents of the round brackets in formula (1); the exact location of these particular ions in the model structures requires further work.

## 8. Summary and conclusions   

New structural–chemical formulae are presented for both single- and double-chain tobermorite-based phases that allow for the presence of vacant tetrahedral sites and substituent ions. Equations are provided that can be used to calculate a number of useful quantities from ^29^Si MAS NMR data, including the fractions of tetrahedral sites that are occupied by Si or Al, or that are vacant.

Chemical and structural data for C-S-H(I) preparations from the literature are collated. The data are consistent with the view that C-S-H(I) which has a Ca/Si ratio < ∼ 1.4 has a structure that is derived from a single-chain tobermorite, but with vacant tetrahedral ‘bridging’ sites. It is shown that there are no interlayer calcium ions when the chains are of infinite length and that one Ca^2+^ ion is added to the interlayer region for each bridging site that is vacant. C-S-H(I) preparations that have a Ca/Si ratio 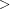 ∼ 1.4 consist of C-S-H(I) intermixed with a Ca-rich phase.

It is not possible to generate a structural model for dimeric C-S-H(I) that is crystal-chemically consistent with known calcium silicate hydrates if the starting structure is an orthotobermorite; *i.e.* of the type that has been used in all previous studies. However, crystal-chemically plausible models can be developed that are instead based on clinotobermorite. A number of models that represent different mean chain lengths are developed and presented. They were derived using crystal-chemical and geometrical reasoning; the necessary crystal-chemical principles were established by inspection of the structures of 35 crystalline calcium silicate hydrates and related phases. These principles should be of use for future structural and atomistic modelling studies.

The models developed in this paper for C-S-H(I) are consistent with experimental observations. In particular, they account for:

(i) The linear decrease in layer spacing that occurs with increasing Ca/Si ratio.
(ii) The decrease in SOF_BT_ that occurs with increasing Ca/Si ratio.
(iii) The presence of dimeric silicate anions at Ca/Si ratios as low as 0.9.
(iv) The observed variation in H_2_O/Si with increasing Ca/Si.
(v) The observed variation in layer spacing with H_2_O/Si ratio.
(vi) The main features on XRD patterns for C-S-H(I). However, use of the developed models in Rietveld refinements will require the acquisition of XRD, ^29^Si MAS NMR and analytical TEM data for the same sample.
(vii) The substitution of Al^3+^ for Si^4+^.

The C-A-S-H(I) preparations that are reported in most studies cannot be taken to represent the C-A-S-H that forms in real cements and the amount of Al that is incorporated into the C-A-S-H in cements appears to represent a practical maximum.

## Supplementary Material

Crystal structure: contains datablock(s) global, T11_14sc, T5_14sc, T5_11sc, T3_14sc, T2_so_LS2, T2_so_LS1, T2_sc_LS2, T2_sc_LS1, T2_ac, Tinf_sc_noCa_LS2, Tinf_sc_noCa_LS1, Tinf_14sc, Tinf_14ac, Tinf_11so, Tinf_11sc, Tinf_11ac. DOI: 10.1107/S2052520614021982/hw5035sup1.cif

CCDC references: 1028747, 1028748, 1028749, 1028750, 1028751, 1028752, 1028753, 1028754, 1028755, 1028756, 1028757, 1028758, 1028759, 1028760, 1028761, 1028762

## Figures and Tables

**Figure 15:**
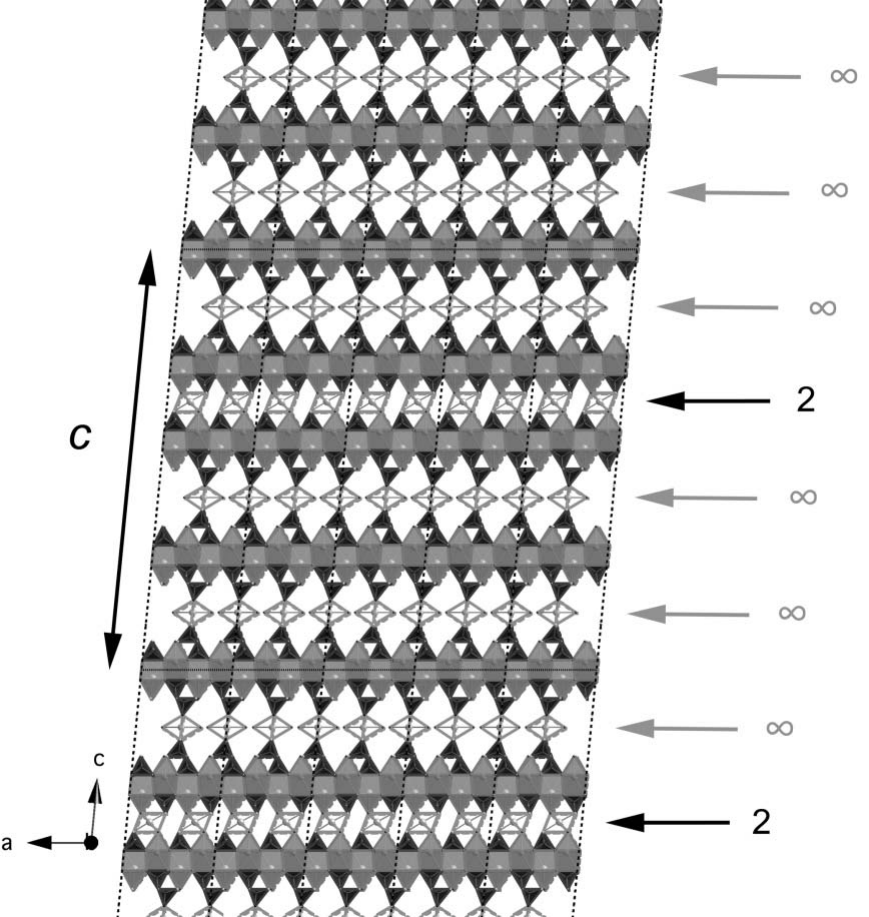
A hypothetical ‘undecamer’ derived from a staggered-chain clinotobermorite, viewed along the **b** axis (edge lengths are *a* and *c*). The T2 and T∞ modules are indicated. The formula is Ca_9_(Si_11_O_28_(OH)_6_)(H_2_O)_7.25_; the space group is *C*1; *a* = 11.35, *b* = 7.3, *c* = 52.7 Å, β = 95.5°; *Z* = 4; calculated density is 2.063 g cm^−3^; *i* = 2 × SOF_BT_ = 1.5. Full details are given in the CIF datablock T11_14sc.

**Table 1:** The fractions of the tetrahedral sites that are present in tobermorites and C-A-S-H phases that are occupied by Si or Al or that are vacant Al substitutes for Si only at bridging sites and paired sites cannot be vacant. The theoretical maximum values of *f*, *v* and (*f* + *v*) are 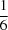, 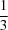 (*i.e.* dimer) and 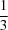, respectively, for double-chain structure and 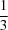, 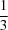 and 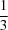 for single-chain structure. The actual minimum and maximum values of *f* are dependent on the fraction (*v*) and distribution of vacant sites.

Sites occupied by	Value† for double-chain tobermorite and cross-linked C-A-S-H	Value for single-chain tobermorite and C-A-S-H
Al	*f*	*f*
Vacancy	*v*	*v*
Q^1^ Si	2*v*	2*v*
Q^2^(0Al) Si	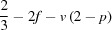	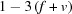
Q^2^(1Al) Si	2*f*	2*f*
Q^3^(0Al) Si	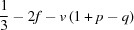	
Q^3^(1Al) Si	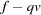	
		
Total	1	1
		
Paired Q^2^(0Al) Si	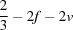	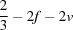
Bridging Q^2^(0Al) Si	*pv*	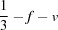
		
Paired Q^2^(0Al) Si adjacent to Q^3^	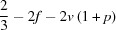	
Paired Q^2^(0Al) Si adjacent to Q^2B^	2*pv*	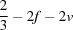
Bridging Q^2^(0Al) Si	*pv*	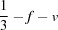

† *p* = the fraction of vacant sites that are adjacent to a bridging site that is occupied by Si; *q* = the fraction of vacant sites that are adjacent to a bridging site that is occupied by Al; it follows that the fraction of vacant sites that are opposite other vacant sites = 1*p* *q*. Vacant sites that are opposite a bridging site that is occupied by Al or that is vacant are illustrated in Fig. 3 ▸ of Myers *et al.* (2013 ▸).

**Table 2:** Values of the variables in literature models for single-chain tobermorite-based C-S-H for the four shortest MCL; it is evident that 
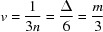 SOF_BT_ is the site occupancy factor for the bridging site [equation (10)] and 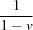 corresponds to the maximum Ca/Si ratio that is possible without the presence of CaOH groups.

MCL		2	5	8	11
This paper	*v*	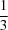	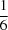	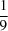	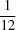
Richardson Groves (1992 ▸)	*n*	1	2	3	4
Taylor (1993 ▸)		2	1	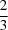	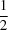
Cong Kirkpatrick (1996 ▸)	*m*	1	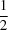	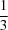	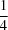
	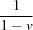	1.5	1.2	1.125	1.091
	SOF_BT_	0	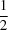	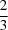	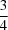

**Table 3:** Selected bond distances () for the model structure for staggered-chain 14 clinotobermorite Full details of the structure are given in the CIF (T_14sc). I = interlayer Ca.

Ca1/1*A*	Ca2/2*A*	Ca3/3*A* (I)		Si1/1*A*	Si2/2*A*	Si3/3*A*
2.387	2.387	2.381		1.599	1.595	1.576
2.387	2.387	2.381		1.612	1.610	1.590
2.400	2.400	2.381		1.614	1.618	1.632
2.400	2.400	2.381		1.660	1.678	1.644
2.560	2.576	2.406				
2.604	2.604	2.406				
2.618	2.618					
2.481	2.483	2.389	Average for polyhedron	1.621	1.625	1.611
	2.454		Overall average		1.619	

## Footnotes

1 C = CaO; A = Al_2_O_3_; S = SiO_2_; H = H_2_O.

2 Supporting information for this paper is available from the IUCr electronic archives (Reference: HW5035).
